# Water-assisted purification during electron beam-induced deposition of platinum and gold

**DOI:** 10.3762/bjnano.15.73

**Published:** 2024-07-18

**Authors:** Cristiano Glessi, Fabian A Polman, Cornelis W Hagen

**Affiliations:** 1 Delft University of Technology, Fac. Applied Sciences, Dept. Imaging Physics, Lorentzweg 1, 2628CJ Delft, Netherlands

**Keywords:** FEBID, gold, nanofabrication, platinum, purification

## Abstract

Direct fabrication of pure metallic nanostructures is one of the main aims of focused electron beam-induced deposition (FEBID). It was recently achieved for gold deposits by the co-injection of a water precursor and the gold precursor Au(tfac)Me_2_. In this work results are reported, using the same approach, on a different gold precursor, Au(acac)Me_2_, as well as the frequently used platinum precursor MeCpPtMe_3_. As a water precursor MgSO_4_·7H_2_O was used. The purification during deposition led to a decrease of the carbon-to-gold ratio (in atom %) from 2.8 to 0.5 and a decrease of the carbon-to-platinum ratio (in atom %) from 6–7 to 0.2. The purification was done in a regular scanning electron microscope using commercially available components and chemicals, which paves the way for a broader application of direct etching-assisted FEBID to obtain pure metallic structures.

## Introduction

Focused electron beam-induced deposition (FEBID) is a nanofabrication technique that allows for the direct writing of three-dimensional nanostructures [1–3]. In FEBID, a gaseous precursor, often an organometallic compound, is injected in the vacuum chamber of a scanning electron microscope (SEM), adsorbed on a substrate, and dissociated by a focused electron beam. As a result, a solid deposit is formed, and organic and inorganic volatile fragments are removed by the vacuum pumps of the chamber. In the case of an organometallic precursor gas, in an ideal scenario, only metal is deposited, and all fragments arising from organic and inorganic ligands are removed. However, in most cases, a large amount of the carbon from the organic ligands is incorporated in the deposits. The removal of the carbonaceous material and, hence, the increase of metallic content of FEBID structures is ultimately important for the performance of the obtained structures in applications such as nanoprobes and sensors [4–6], or for the fabrication of superconductors [7–10] and plasmonic devices [11–13]. A lower carbon content can be achieved by means of precursor design [14], variation of deposition conditions [15], or post-deposition purification [16]. One of the most widely used FEBID gaseous precursors is trimethyl(methylcyclopentadienyl)platinum(IV) (MeCpPtMe_3_), which, under standard deposition conditions, leads to the deposition of a material that consists of around 15 atom % Pt, with the rest of the material consisting of an amorphous carbon matrix [15,17–18]. Variation of deposition conditions [15] and post-deposition purifications [18–21] have all led to an increased Pt content in the deposits, but a straightforward method of obtaining pure material is still a challenge. However, Shawrav et al. [22] managed to deposit pure gold structures in a single process step using the co-injection of the precursor Au(tfac)Me_2_ and water. This inspired the present work, in which we aim for the direct deposition of high-purity Au and Pt nanostructures achieved through the co-injection of water and the precursors Au(acac)Me_2_ and MeCpPtMe_3_ respectively.

### Purification of FEBID materials with a reactant gas agent

Purification of FEBID products by the use of a reactant gas has been performed either through a one-step procedure, with the co-injection of the FEBID precursor and the reactant gas (purification during deposition), or through a two-step procedure, with an initial deposition from the deposition precursor and a successive purification in the presence of a reactant gas (post-deposition purification). Several reactant gases have been tried, either in pure form, such as water [22–25], oxygen [18,26–27], hydrogen [26–27], and ammonia [28–29], as a mixture of argon and oxygen [30–31], or in an oxygen plasma [32–33]. The success of these purification attempts varies considerably depending on the chemical nature of the precursor and the reactant gas. When using an oxidant gas reactant, such as water or oxygen, during electron beam exposure, successful purification of the deposited material occurs only if the deposited metal is inherently resistant to oxidation, such as Au, Pt, and Ru [34]. In the case of iron [35], the removal of carbon is accompanied by a large incorporation of oxygen in the deposit, resulting in the fabrication of metal oxide nanostructures. Post-deposition purification requires the removal of carbon after completion of the deposition, causing porosity and/or severe changes in size and shape. In addition, post-deposition purification may lead to only partly purified material. For example, post-deposition treatment using O_2_ as oxidizing gas (in combination with electron-beam exposure) of PtC*_x_* material deposited from MeCpPtMe_3_ resulted in the purification of the top surface of the deposit only [36]. In contrast, using water as the oxidizing gas (in combination with electron beam exposure) led to the purification of a 40 nm thick bottom layer of the deposit [37–38]. Fully post-deposition-purified FEBID structures were reported by Seewald et al. [4], who deposited three-dimensional hollow cones of moderate thickness, which were purified using an e-beam-assisted post-deposition process in a low-pressure (80 Pa) water environment. Interestingly, deformation caused by electron beam-induced post-deposition purification can intentionally be used to bend the three-dimensional structure of FEBID deposits [39]. Purification during deposition avoids some of the disadvantages of post-deposition purification because of the concomitant deposition and purification, which should lead to the direct deposition of the desired FEBID structures. Furthermore, deposition and purification at the same time are expected to produce pure bulk material [24]. In Table 1 an overview of the currently reported attempts of electron beam-assisted purification of Au and Pt deposits using an oxidant gas reactant is presented.

**Table 1:** Electron beam-assisted purification of Au and Pt FEBID materials with oxidant gas reactants.

Precursor	Purification time	Type of purification	Metal content (atom %)		Ref.
	
			unpurified	purified	

MeCpPtMe_3_	post-deposition	O_2_, 7·10^−6^ mbar, 200 °C	C/Pt 1.0–1.1^a^	C/Pt 0.12^a,b^	[19]
MeCpPtMe_3_	post-deposition	Ar/O_2_, 7^.^10^−6^ mbar	C/Pt 2^a^	pure metal^b^	[20]
MeCpPtMe_3_	post-deposition	O_2_, 4^.^10^−5^ mbar	C/Pt 1.0^a^	C/Pt 0.1^a^	[21]
MeCpPtMe_3_	post-deposition	O_2_, 10^−5^ mbar, laser	PtC_5_	pure metal^b^	[40]
MeCpPtMe_3_	post-deposition	O_2_, 10^−5^ mbar	PtC_5_	pure metal surface^b^	[36]
MeCpPtMe_3_	post-deposition	H_2_O, 0.1 mbar	C/Pt 1.9^a^	pure metal^b^	[37]
MeCpPtMe_3_	during deposition	O_2_, 10^−4^ mbar	C/Pt 1.0^a^	C/Pt 0.1^a^	[21]
MeCpPtMe_3_	during deposition	H_2_O, 8·10^−6^ mbar	15	50	[23]
Pt(PF_3_)_4_	during deposition	O_2_, 10^−5^ mbar, hot substrate 80 °C	15	circa 20	[41]
Au(acac)Me_2_	post-deposition	O_2_, 5·10^−5^ mbar	C/Au 0.45^a^	C/Au 0.05^a,b^	[42]
Au(acac)Me_2_	post-deposition	O_2_, 10^19^ particles/cm^2^·s	C/Au 0.6^a^	C/Au 0.06^a,b^	[43]
Au(acac)Me_2_	during deposition	H_2_O, 1.2 mbar	—	solid gold core	[24]
Au(acac)Me_2_	during deposition	O_2_, 5·10^−5^ mbar	C/Au 0.45^a^	C/Au 0.1^a,b^	[42]
Au(acac)Me_2_	during deposition	H_2_O, 1 mbar	80 wt %	60 wt %	[25]
Au(tfac)Me_2_	post-deposition	oxygen plasma	30	72	[32]
Au(tfac)Me_2_	post-deposition	H_2_O, 2·10^−4^ mbar	43	76	[44]
Au(tfac)Me_2_	post-deposition	oxygen plasma	C/Au 0.9^a^	C/Au 0.34^a^	[33]
Au(tfac)Me_2_	during deposition	H_2_O, 2·10^−4^ mbar	30	91	[22]
Au(hfac)Me_2_	during deposition	Ar/O_2_, 13 mbar	25	50	[30–31]

^a^EDX peak intensity ratio; ^b^Pure metal is achieved when the EDX peak intensity ratio C/metal reaches circa 0.1.

Ammonia has been also applied as a gas reactant for post-deposition purification of ruthenium FEBID deposits, causing the substitution of the deposited carbon with nitrogen. No purification was achieved, but the functionalization of the material could lead to interesting applications, such as the production of nitrides using FEBID [28].

### Water-assisted reactions in Pt FEBID deposits

**Figure 1:**
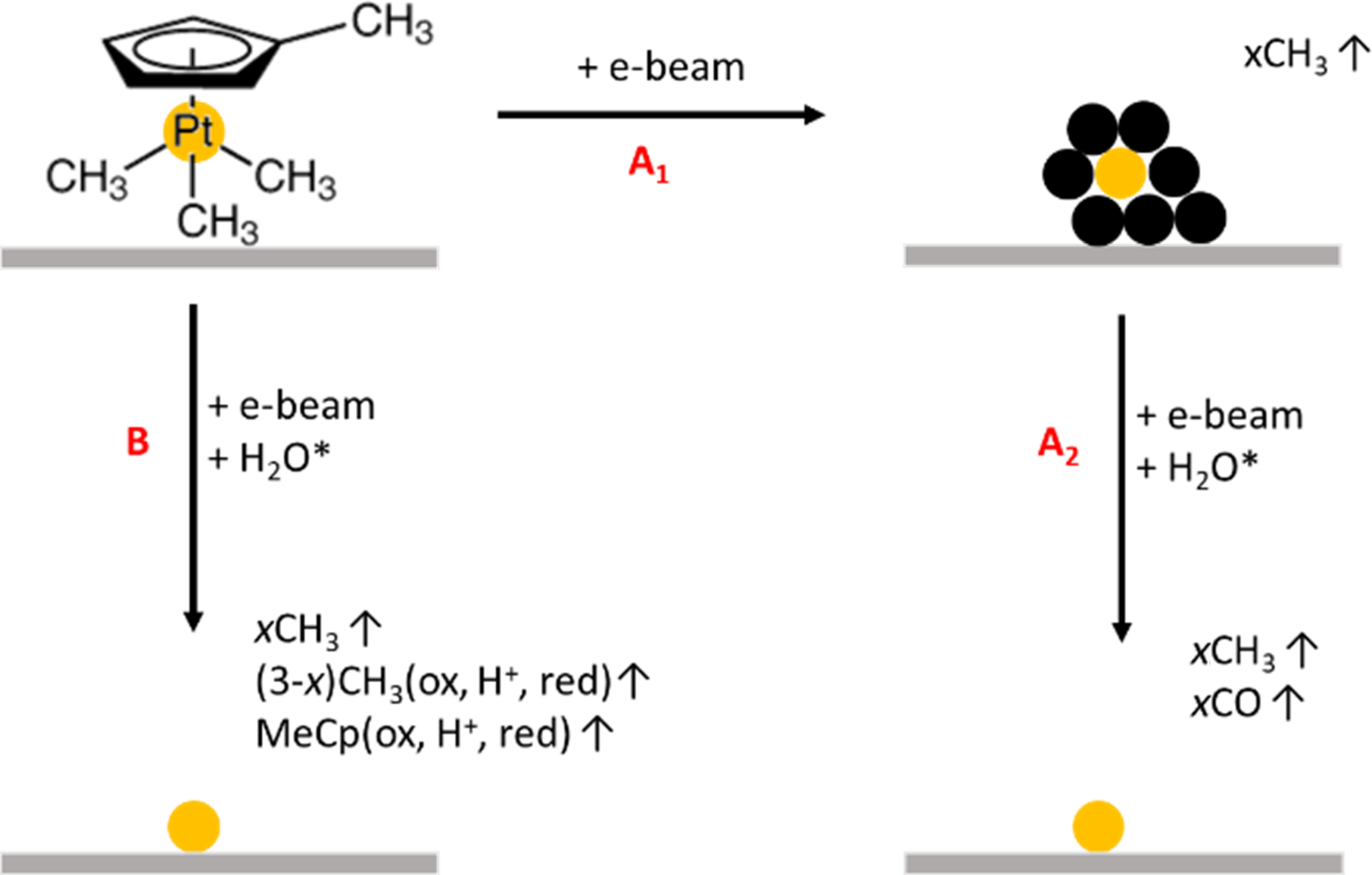
Extreme cases for water-assisted purification pathways during e-beam deposition of MeCpPtMe_3_. (A_1_) e-Beam deposition and creation of PtC*_x_* with (A_2_) subsequent water-assisted purification. (B) Concomitant e-beam deposition and water-assisted purification with removal of most ligands as oxidized (ox), reduced (red), or protonated (H^+^) forms of the ligands.

Water-assisted post-exposure purification of condensed layers of MeCpPtMe_3_ that mimic Pt FEBID deposits was monitored in ultrahigh vacuum by mass spectrometry, leading to the detection of both gaseous CO and CH_4_ species [45]. The production of these two volatile species is ascribed to two different processes: (i) for CH_4_ the removal of trapped species and (ii) for CO the electron-induced hydration of double bonds of MeCp (or derived fragments) incorporated in the deposit and the consequent production of alcohols, which are likely to produce CO during electron exposure [45–47]. This observation was substantiated by the increase of desorbed CO throughout exposure, indicating the presence of an intermediate step in the production of CO. These findings could be extended to purification during deposition for cases in which, in a first step, PtC*_x_* is deposited, and, in a second step, the carbon from the partially dissociated parent molecules is removed by oxidation (Figure 1, pathway A1, A2). However, a different purification mechanism is possible since organic ligands could be removed from adsorbed precursor molecules by the electron-activated water species and cause a favourable decomposition of the adsorbed material (Figure 1, pathway B).

Radiolysis of water can generate acid species, such as H_3_O^+^, that are known to cause protonation of (methyl)cyclopentadienyl to (methyl)cyclopentadiene (MeCpH). MeCpH is weakly bonded to the metal and is easily removed from the Pt centre [48–49]. This decomposition pathway was not directly observed in post-deposition purification studies, but could be more accessible in purification during deposition since the amount of intact MeCpPtMe_3_ molecules adsorbed to the substrate should be larger.

Similarly, the protonation of an acetylacetonate ligand to form acetylacetone causes the formation of a much weaker metal–ligand interaction. Such an acetylacetonate ligand is present in the complex Au(acac)Me_2_, and its protonation could be a possible fragmentation pathway for the complex.

## Experimental

### General

Deposition experiments were performed in a Thermo Fisher Scientific (TFS) Nova Nano Lab 600i dual-beam system. Standard TFS gas injection systems (GISs) were used for both the deposition and etching (water) processes. The Au deposition precursor used is Au(acac)Me_2_, the Pt deposition precursor is MeCpPtMe_3_, and the etching precursor is MgSO_4_·7H_2_O. The GIS temperatures used were 24 °C for Au(acac)Me_2_ and MeCpPtMe_3_; for MgSO_4_·7H_2_O, the temperature varied between 24 and 35 °C for Au deposition, and between 30 and 35 °C for Pt deposition. The pressure increase in the SEM chamber is taken as a measure of the water flux. The latter is controlled through the temperature setting of the GIS reservoir and manual adjustment of the GIS valve. Before deposition and after loading the sample, the chamber was plasma-cleaned with an XEI Scientific Evactron decontaminator for 30 min, at 0.4 Torr (air leak) and a forward radio frequency power of 12 W. Silicon substrates were obtained from a 525 ± 25 µm p-type silicon wafer (resistivity of 1–5 Ω·cm, (100) crystal orientation) with a native silicon oxide layer. Samples of 1 × 1 cm^2^ were used, on to which an array of annular patterns was lithographically defined, by laser lithography and etching using an SF_6_–O_2_ dry-etch, to facilitate location of the deposition areas. The substrates were roughly cleaned in acetone and subsequently sonicated for 2 min in acetone and for 2 min in isopropyl alcohol. All depositions were performed with both GIS nozzles inserted. Deposition, imaging, and EDX analysis were performed in immersion mode at a working distance of 5 mm.

### Deposition conditions

In general, the main parameters that control the deposition are the precursor supply, the primary beam energy and current, and the patterning strategy. The deposits are built through consecutive electron beam spot exposures of the precursor molecules adsorbed on the substrate. The shape of the deposit is defined as an area containing an array of discrete exposure points. The distance between exposure points is the pitch, while the exposure time for each point is the dwell time. This pattern is repeated for a certain number of passes [1].

The shape and size of the deposits are defined using the TFS “rectangle” or “line” patterning tools. The main patterning parameters are the patterned area size, dwell time, primary beam energy and current, pitch, number of passes, and SEM chamber pressure during deposition or chamber pressure increase during deposition. The complete parameters for the deposits presented in this work are presented in Supporting Information File 1. For all experiments, the primary beam energy was kept between 5 and 18 kV, and the pitch was kept at 4 nm. The chamber background pressure was always circa 1·10^−6^ mbar, unless otherwise specified. In the case of series with a variation of one of the parameters, the specific values are discussed further. All deposits presented are patterned under co-injection of deposition and etching precursors, unless otherwise specified.

#### Gold deposits

A series of rectangular 500 × 500 nm^2^ gold deposits were deposited while varying the pressure increase in the SEM chamber upon the injection of the water precursor in the range of 5.3·10^−7^–4.0·10^−5^ mbar. Au and H_2_O GIS nozzles were positioned equidistantly from the deposition area at a distance of 125–130 µm above the substrate and circa 200 µm from the beam position.

#### Platinum deposits

The Pt and H_2_O GIS nozzles were positioned in two different configurations, that is, the water GIS was positioned 150 µm above the substrate and 100 µm from the beam position, while the Pt GIS nozzle was positioned 0.54 mm above the substrate and 1.29 mm from the beam position.

**Pressure variation series:** A series of rectangular platinum deposits was deposited while varying the pressure increase in the SEM chamber upon the injection of the water precursor in the range of 0–4.7·10^−5^ mbar.

**Current variation series:** A series of rectangular platinum deposits was deposited while varying the current of the primary beam (deposits indicated by **1b**–**1e**) in the range of 0.13–2.70 nA. In addition, a longer deposition experiment was performed for better EDX quantification at 0.54 nA (**1a**). Additional experiments include a Pt-only deposit for reference (**1f**) and a sample prepared for TEM analysis (**1g**). Except for **1f**, all deposits were obtained at a deposition pressure of (4.6–5.7)·10^−5^ mbar.

**Purification process investigation:** Sets of nine rectangular Pt deposits were patterned with increasing number of passes at two different beam currents (0.54 and 2.3 nA), each set with either co-injection of Pt and H_2_O precursors, injection of Pt precursor only, or injection of H_2_O precursor only (**2a**–**2f**). Also, a line pattern was deposited and analysed (**2g**).

### EDX

EDX measurements were performed in the same instrument using an Oxford Instruments X-MAX 80 detector. The working distance was kept at 5 mm to have the optimum EDX signal. All spectra were analysed using the Oxford Instruments AZtec software. All EDX spectra were recorded at 5 keV. The C/metal atomic percentage ratio, with either gold or platinum metal, is used as a representative measure of the purity of the deposits. All C/Pt ratios presented are not corrected for the Pt N peak, which almost coincides with the C K peak, unless specifically mentioned. A correction of this peak using reported values (Pt N/Pt M intensity ratio of 0.09) can be applied by lowering the C/Pt ratio accordingly [20]. As such, we consider a C/Pt atom % ratio of circa 0.26 as an indication of pure Pt. A more extensive explanation of the C/Pt atom % ratio correction can be found in Supporting Information File 1 (pp S3–S5).

### TEM

A TEM lamella was prepared in a TFS Helios dual-beam system. A PtC*_x_* layer was deposited from MeCpPtMe_3_ as a protection layer. Transmission electron microscopy (TEM) and STEM-EDX analysis were performed in an FEI Titan Cubed Cs-corrected TEM at 300 keV. STEM-EDX was performed with a Thermo Fisher Scientific EDX super-X detector in the ChemiSTEM configuration. For EDX mapping, three-pixel averaging was used.

## Results and Discussion

### Water-assisted purification of gold deposits

In order to validate the compatibility of the protocol for water-assisted purification during deposition, as reported in [22], with the deposition conditions achievable in the Nova Nano Lab dual-beam system, first deposition experiments of gold were performed from the Au(acac)Me_2_ precursor with and without water injection during deposition. Purification of the gold deposits in [22] was achieved using a two-nozzle system similar to the one described in the Experimental section, a highly tuneable system that allows for the independent control of the etching and deposition gas fluxes, as also demonstrated in the oxygen-assisted purification of platinum deposits [50–51].

**Figure 2:**
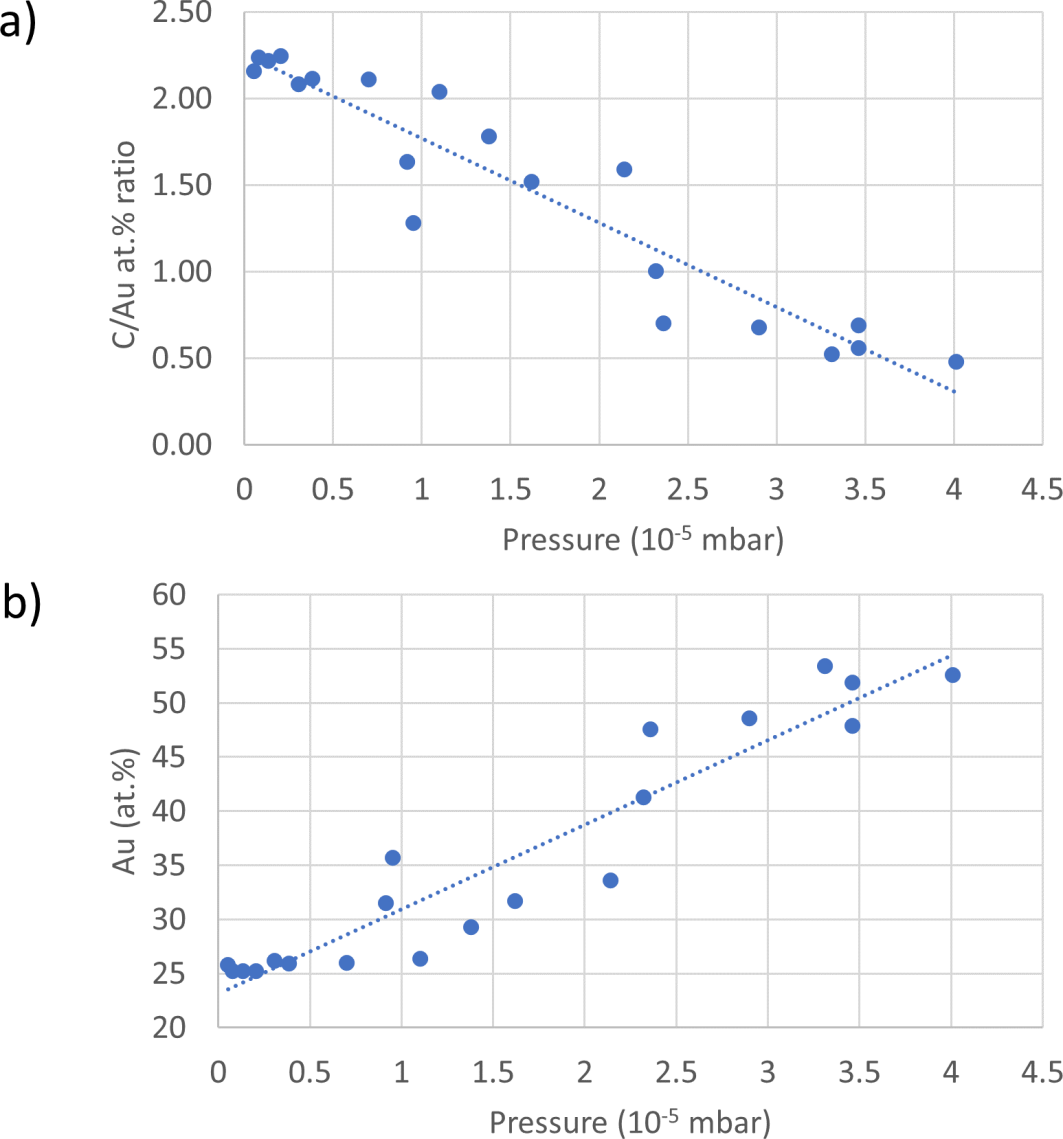
(a) C/Au atom % ratio and (b) Au content in atom % as function of the chamber pressure increase due to water injection. The Au atom % was obtained by quantifying the EDX signals of Au, C, O, and Si.

The reservoir of the Au precursor was kept at room temperature (24 °C), and deposits were made while varying the water reservoir temperature from 24 to 35 °C, to increase the water flux. The increase of the chamber pressure is used as a measure for the increasing water flux. At each chamber pressure, the composition of the deposit was determined through EDX analysis. The carbon/metal atomic percentage ratio (C/M atom % ratio) reflects the amount of purification, in terms of carbon removal, with increasing water flux. A C/M ratio of 1 means that for each metal atom one carbon atom is deposited. In a fully purified structure, the C/M atom % should be zero. The precursor molecule Au(acac)Me_2_ contains seven carbon atoms for each gold centre, leading to a C/M atom % ratio of 7. The deposited material, without any water injection, was found to have a C/Au atom % ratio of 2.8. This value is in the lower range of the C/Au atom % values reported for the deposition from Au(acac)Me_2_ in the absence of any purification (circa 3–12.7) [12,52–54]. The low value of the C/Au ratio could be due to a favourable fragmentation, perhaps as a result of the rather low precursor flux used; but it could also be the result of the presence of some residual water in the SEM chamber. Nevertheless, when increasing the water flux, a clear decrease of the carbon content of the deposits is observed with the corresponding chamber pressure increase, following a linear trend as seen in Figure 2a. The maximum gold content achieved is 52 atom % at a chamber pressure of 4·10^−5^ mbar (Figure 2b). This result is even higher than the value reported by Shawrav et al., who obtained ca. 46 atom % Au from Au(tfac)Me_2_ at the same chamber pressure [22]. Unfortunately, higher water pressures could not be explored in this study. The limiting factor to the achievable pressure in the SEM chamber and, hence, water injection is the rapid increase of the pressure in the mid-column when the main chamber pressure rises above 5·10^−5^ mbar. At such pressures, the electron column valve is automatically closed. The present result shows that the water-induced purification during the deposition of gold is not limited to fluorinated gold acetylacetonate-based precursors. Furthermore, it expands on the possibility of purifying FEBID material deposited from Au(acac)Me_2_ as a precursor using water as oxidizing agent under non-environmental SEM conditions [24].

### Water-assisted purification of platinum deposits

**Figure 3:**
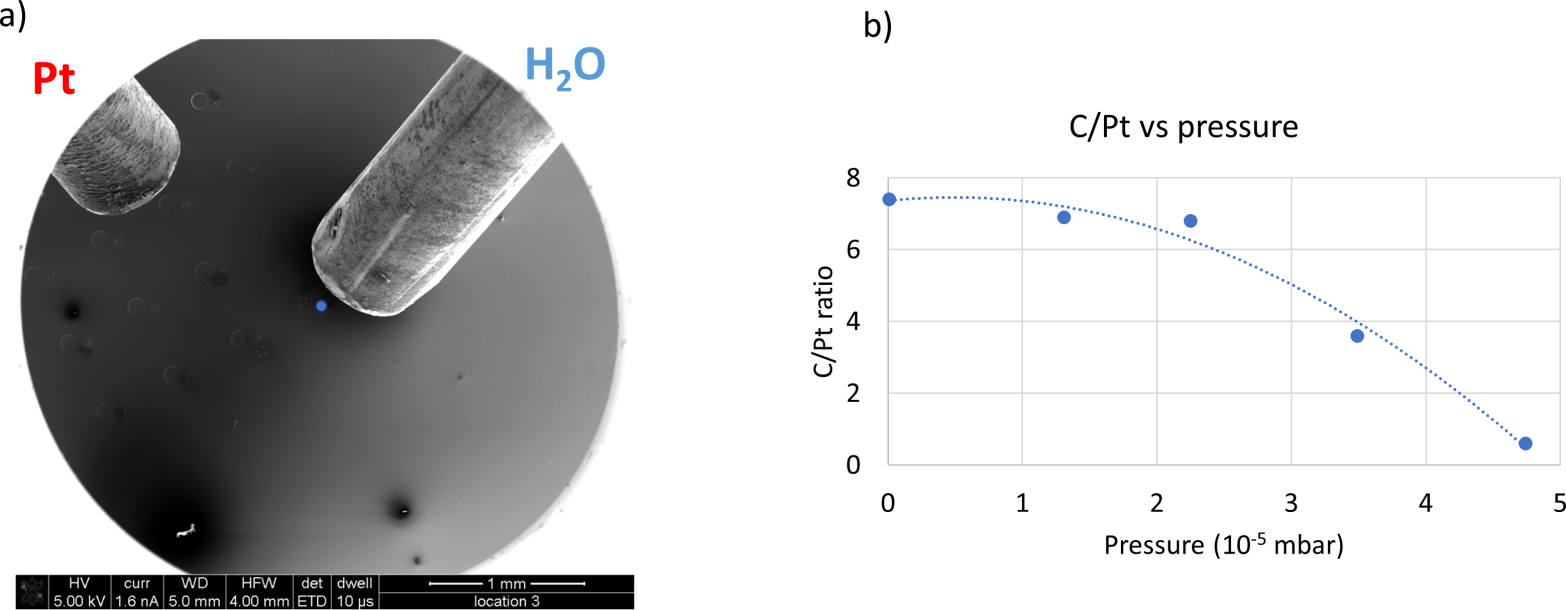
(a) Positioning of the water GIS (right) and the Pt GIS (left), the blue dot indicates the deposition area. (b) Variation of the C/Pt atom % ratio as a function of the chamber pressure increase during deposition (see also Supporting Information File 1, pp S11–S13).

Purification of Pt deposits was achieved only by using co-injection from two separate nozzles. Experiments with co-injection of both gases from the same nozzle led to no purification of the deposits. Also, only when injecting water after the injection of the Pt precursor, purification was achieved. In that case, Pt precursor adsorbed on the inner surface of the nozzle was carried by the subsequent water flux and deposited. Unfortunately, these experimental conditions cannot be very well controlled. However, these experiments did indicate the importance of a high gas flux ratio H_2_O/MeCpPtMe_3_ to achieve purified deposits. A more extensive presentation of these data is provided in Supporting Information File 1 (pp S8–S10). In the two-nozzle system, the gas flux ratio was maximized by positioning the nozzle of the water GIS 150 µm above the substrate and 100 µm from the beam position, while the Pt GIS nozzle was at a much larger distance from the deposition area, that is, 0.54 mm above the substrate and 1.29 mm away from the beam position (Figure 3). Furthermore, the Pt precursor was kept at room temperature (24 °C) to minimize the precursor flux. Because of the low vapour pressure of the Pt precursor at 24 °C and the remote position of the Pt GIS nozzle, the deposition of platinum is limited by adsorption and surface diffusion of the precursor molecules [51]. A high water flux was necessary for the purification of the deposits to compensate for the low electron-stimulated dissociation cross section of water on the substrate [37]. Deposits were made at increasing water flux, indicated by an increase of the total chamber pressure. The carbon and platinum contents were determined afterwards, and the C/Pt atom % ratio is plotted in Figure 3b as a function of the chamber pressure increase. When no water is injected, the C/Pt atom % ratio is 7.4, indicating roughly the removal of up to two methyl ligands on average during deposition. Up to a chamber pressure of 2.3·10^−5^ mbar, no appreciable purification is observed; the C/Pt ratio is 6.8. At a pressure of 3.6·10^−5^ mbar, the C/Pt ratio has decreased to 3.6, indicating that the Cp ring moiety has been removed at least partly. At the maximum achievable chamber pressure of approx. 5·10^−5^ mbar, a C/Pt ratio of 0.6 is obtained. At this water pressure, the deposited material is already comprised mostly of Pt as for each deposited metallic centre only 0.6 carbon atoms are incorporated (Figure 3). The limiting factor to the water injection is the maximum allowable pressure in the SEM chamber, which also leads to a maximum deposition time of about 10 min for each experiment at 5·10^−5^ mbar. A GIS simulation tool [55] was used to estimate the flux of the two precursors when purification was achieved. An injection flux of water 33 times higher than that of platinum precursor was calculated, which increases to 430 in the deposition area because of the different injection distances.

The order in which the gases were introduced into the chamber before the deposition was started did not have an effect on the composition of the deposited material. Both in the case of the Pt GIS being opened 2 min prior to the water GIS and in the reverse case, the same shape and composition of the deposit are obtained (Supporting Information File 1, pp S14–S15). The relatively large distance of the Pt GIS nozzle from the deposition area, combined with the low Pt precursor flux (low reservoir temperature), leads to a small growth rate. To test whether the deposition and etching processes occur simultaneously, deposits with increasing deposition time (obtained by increasing the number of passes) were produced. If the two processes are simultaneous and Pt precursor is supplied throughout the exposure time, an increase in deposit thickness is expected. Such increase was observed by a decrease of the Si content in the EDX spectrum, which indicates that the two processes occur at the same time (Supporting Information File 1, pp S14–S15). This effectively rules out the possibility of a post-deposition purification process, in which only the adsorbed Pt precursor is deposited in the first few seconds of exposure followed by purification only in the remaining exposure time.

When a PtC*_x_* structure is exposed only to water, that is, when there is no electron exposure, no purification of the structure is observed. This means that cross-purification during subsequent experiments on the same substrate will not occur. Even if a second structure in close proximity is exposed to electrons during water injection, no additional purification is observed in the unexposed PtC*_x_* structure (Supporting Information File 1, pp S16–S17).

**Figure 4:**
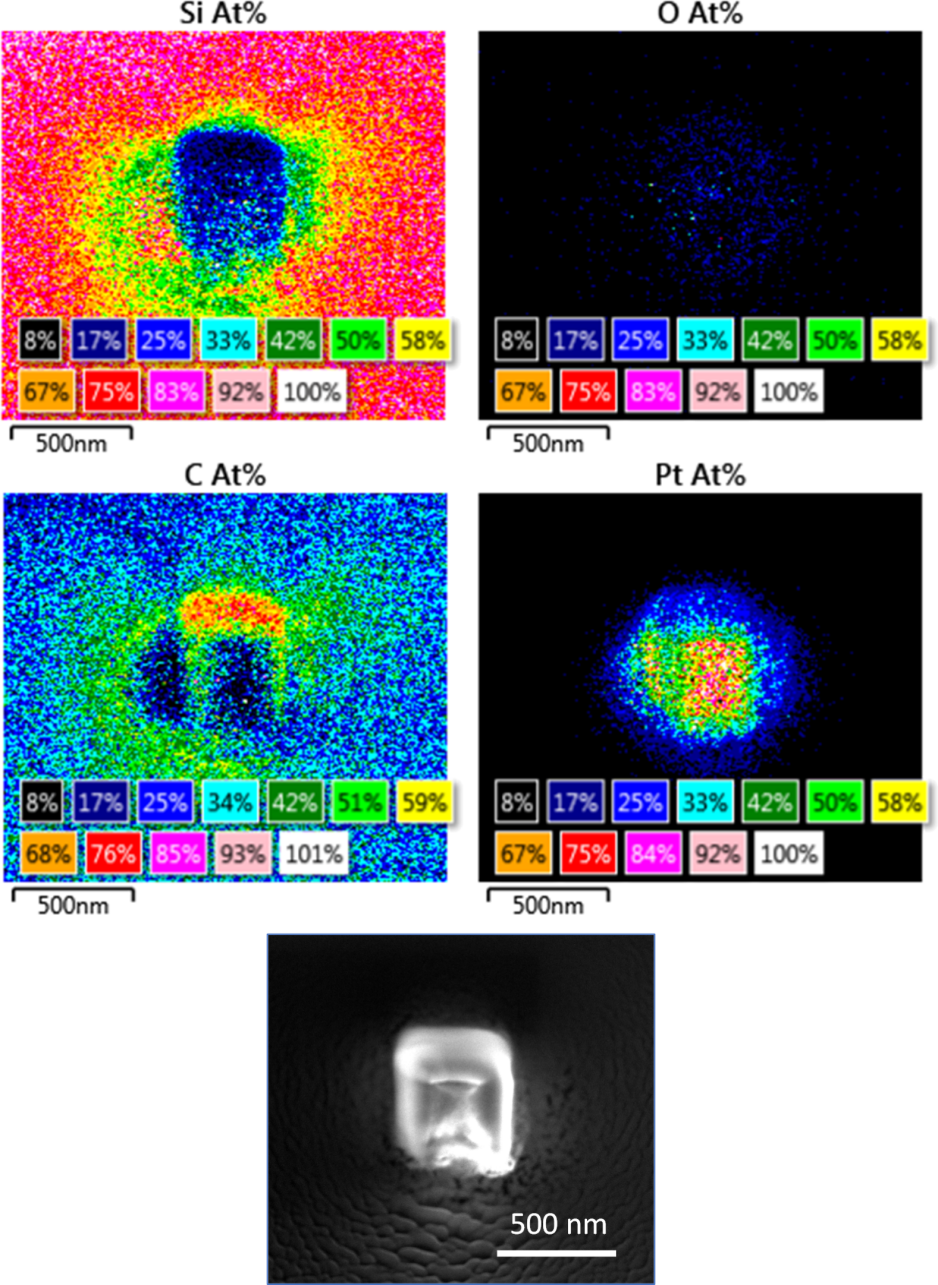
EDX maps for Si, C, Pt, and O presented in atom % for **1a** (colour images generated by the Aztec software; 1% rounding error on the C atom % map). The bottom image is a secondary electron SEM image of the deposit.

In order to obtain a more reliable EDX signal from the deposited material, a large square deposit of 400 × 400 nm^2^ was grown at 5 kV and 0.54 nA with 400,000 passes. It is clear from the EDX maps of the deposit (Figure 4) that the Pt signal is present only in the patterned area, while the carbon signal is visible as a halo around the patterned area. The largest amount of carbon, even reaching the carbon content of an unpurified Pt deposit, is seen on the sides of the deposit oriented towards the GIS nozzles (the GIS nozzle positions are as presented in Figure 3a). While this is attributed to the high availability of precursor in an area of low electron flux, the unavoidable concomitant deposition of hydrocarbons supplied by surface diffusion cannot be completely ruled out. From the carbon EDX map it is also interesting to observe that the C signal is more intense on the unexposed substrate than in the patterned area. Oxygen is present only in limited quantities throughout the patterned area and the halo region. Point EDX performed in the centre of the structure gave a Pt content of 64.1 atom % and a C/Pt atom % ratio of 0.23. Only 2.2 atom % oxygen is incorporated in the deposit, indicating no serious oxidation of the deposited Pt.

**Figure 5:**
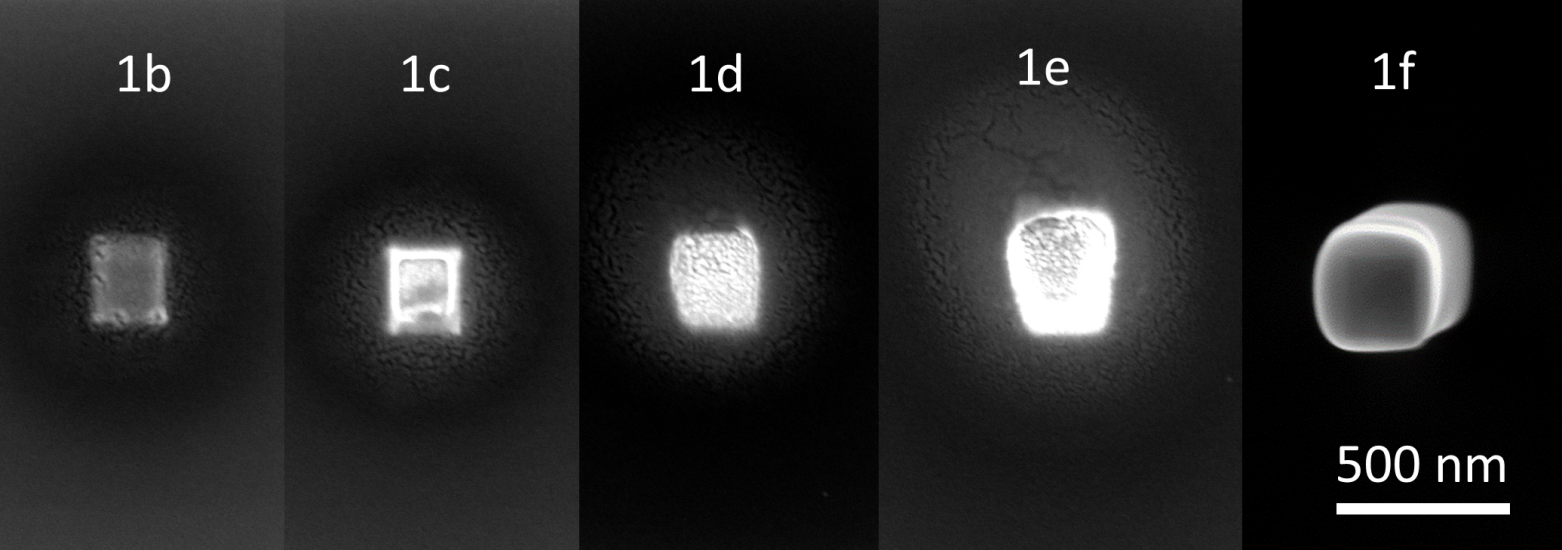
SEM image of deposits **1b**–**1f** obtained by co-injecting platinum precursor and water. All images are taken at the same brightness and contrast settings for clear comparison. Deposits **1b**–**1e** are ordered by increasing electron current. Deposit **1f** is a reference deposit obtained by the injection of only the Pt precursor. The deposition parameters are reported in the Experimental section.

**Figure 6:**
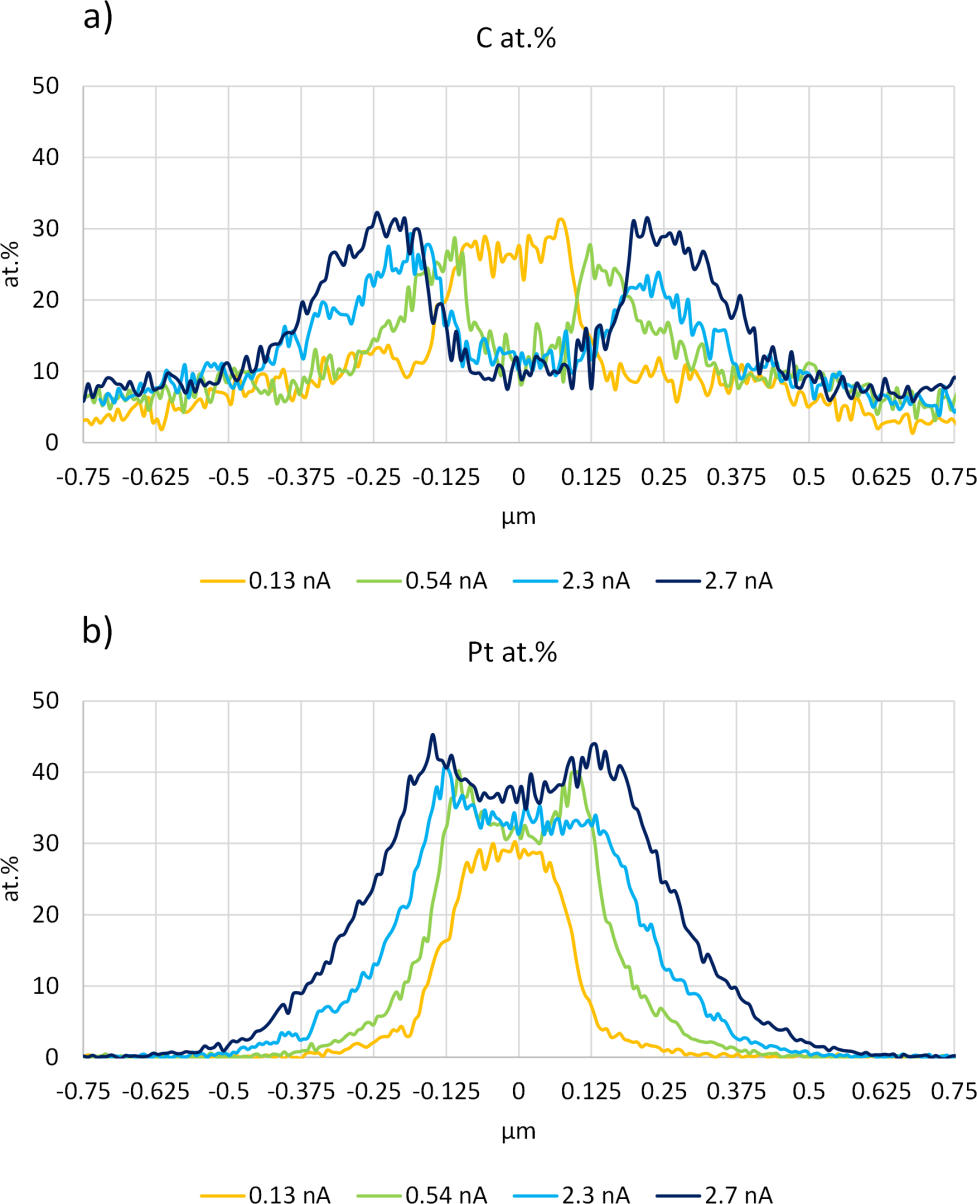
Horizontal line EDX of deposits **1b**–**1e**. The patterned area ranges between −0.125 and 0.125 µm. The (a) carbon and (b) platinum contents are presented in atom %. The background Si signal was not excluded from the analysis.

The difference in composition inside and outside the patterned area can only be associated with the difference in local electron flux since water and Pt precursor fluxes do not vary much over both regions. To study the dependence of the composition of the deposits on the beam current, the current was varied while keeping all other parameters constant. In the explored current range between 0.13 and 2.70 nA, a large change of carbon incorporated in the patterned area can be observed when going from 0.13 to 0.54 nA, indicating that a switch from partial to full purification takes place (see Table 2). In the SEM images of Figure 5 and in the corresponding line-scan EDX spectra of Figure 6, it is seen that this switch is accompanied by the beginning formation of the halo outside of the patterned area, resulting from the higher availability of secondary electrons generated by the backscattered electrons (BSEs). When the current is increased further, the halo becomes larger, and the maximum carbon content is found further away from the patterned area. This indicates that, at higher currents, the increased electron flux in the halo region also progressively purifies the halo. Alternative methods to increase the electron flux while keeping the precursor supply constant, such as increasing the dwell time [56], were not explored further.

**Table 2:** Comparison of point EDX composition (atom %) of the patterned area for deposits **1b**–**1f** and C/Pt atom % ratio. The full spectra are presented in Supporting Information File 1, pp S18–S28.

Deposit	Current (nA)	C	O	Si	Pt	C/Pt	Pt without Si

**1b**	0.13	27.5	4.3	39.9	28.3	0.97	47.1
**1c**	0.54	12.5	7.3	49.3	30.9	0.40	61.0
**1d**	2.3	11.8	6.1	49.3	32.9	0.36	64.8
**1e**	2.7	11.4	9.0	38.9	40.7	0.28	66.6
**1f** ^a^	0.54	78.8	1.1	8.3	11.8	6.7	12.9

^a^reference material, Pt injection only.

**Figure 7:**
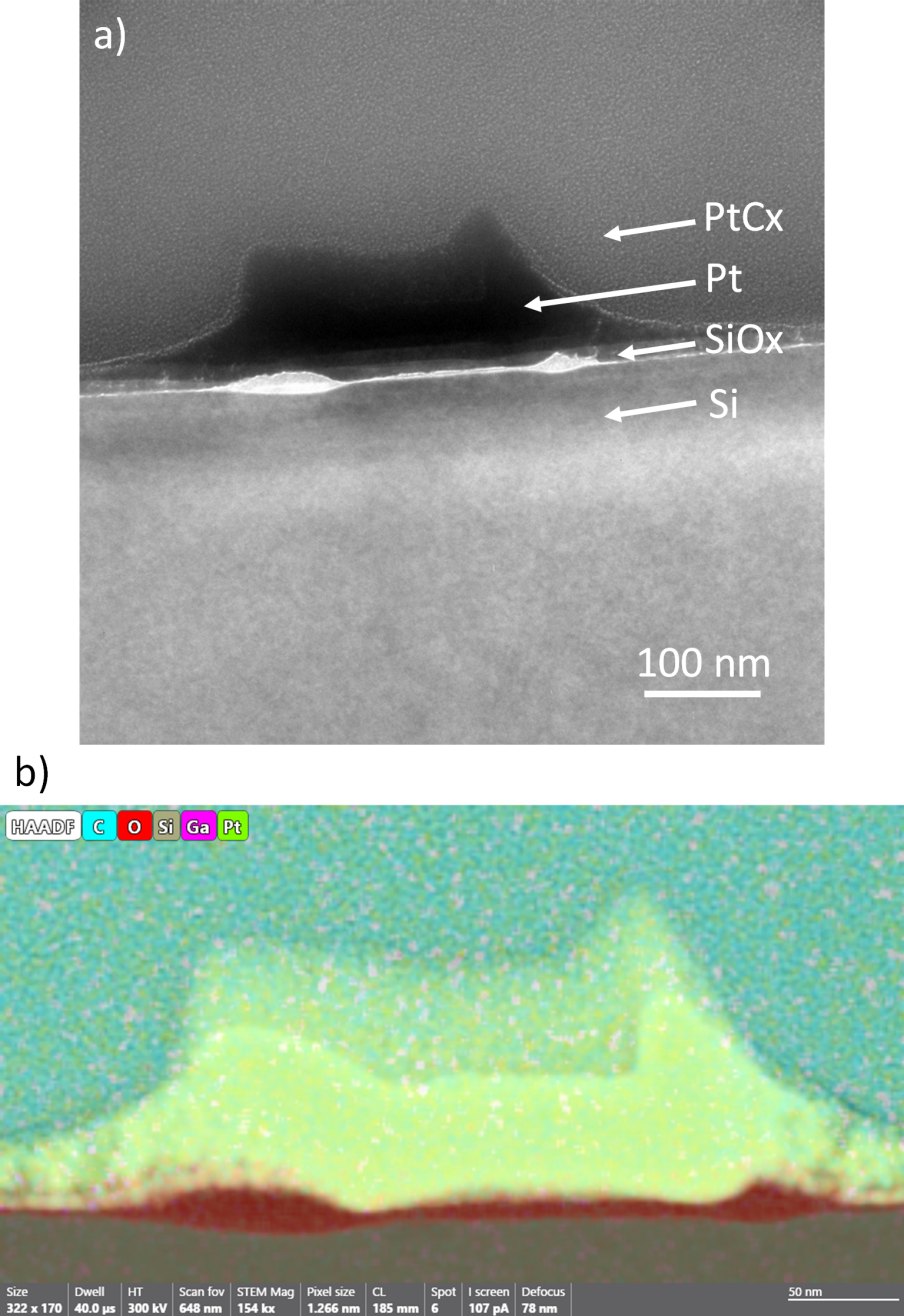
(a) High-resolution TEM image and (b) overlay of the HAADF image and the STEM-EDX map of the cross section of deposit **1g**. Layers from bottom to top: Si substrate, layer of SiO*_x_* formed at the interface between the Si bulk material and the deposited Pt, deposited Pt, partially purified halo, and PtC*_x_* protection layer.

The composition of the material was confirmed by STEM-EDX of a lamella cut from deposit **1g**. The C/Pt atom % ratio in this deposit is consistently in the range of 0.22 ± 0.016, slightly lower than the SEM-EDX top-down line-scan ratio of 0.5 (Supporting Information File 1, p S29) obtained on the same deposit. In the TEM images and the STEM-EDX maps (Figure 7, Supporting Information File 1, pp S30–S31), three layers can be distinguished: (i) the SiO*_x_* layer produced through water-induced oxidation of the Si substrate, (ii) the Pt layer deposited in the patterned area (with a height of circa 60 nm), and (iii) the partially purified halo around the patterned area, which appears on top of the purified area in the lamella projection (Figure 7b, Supporting Information File 1, pp S30–S31). The SiO*_x_* layer is thickest (19 nm) in the area surrounding the patterned area, while it is thinner (6 nm) directly under the patterned area.

### Insights in the purification process during deposition

**Figure 8:**
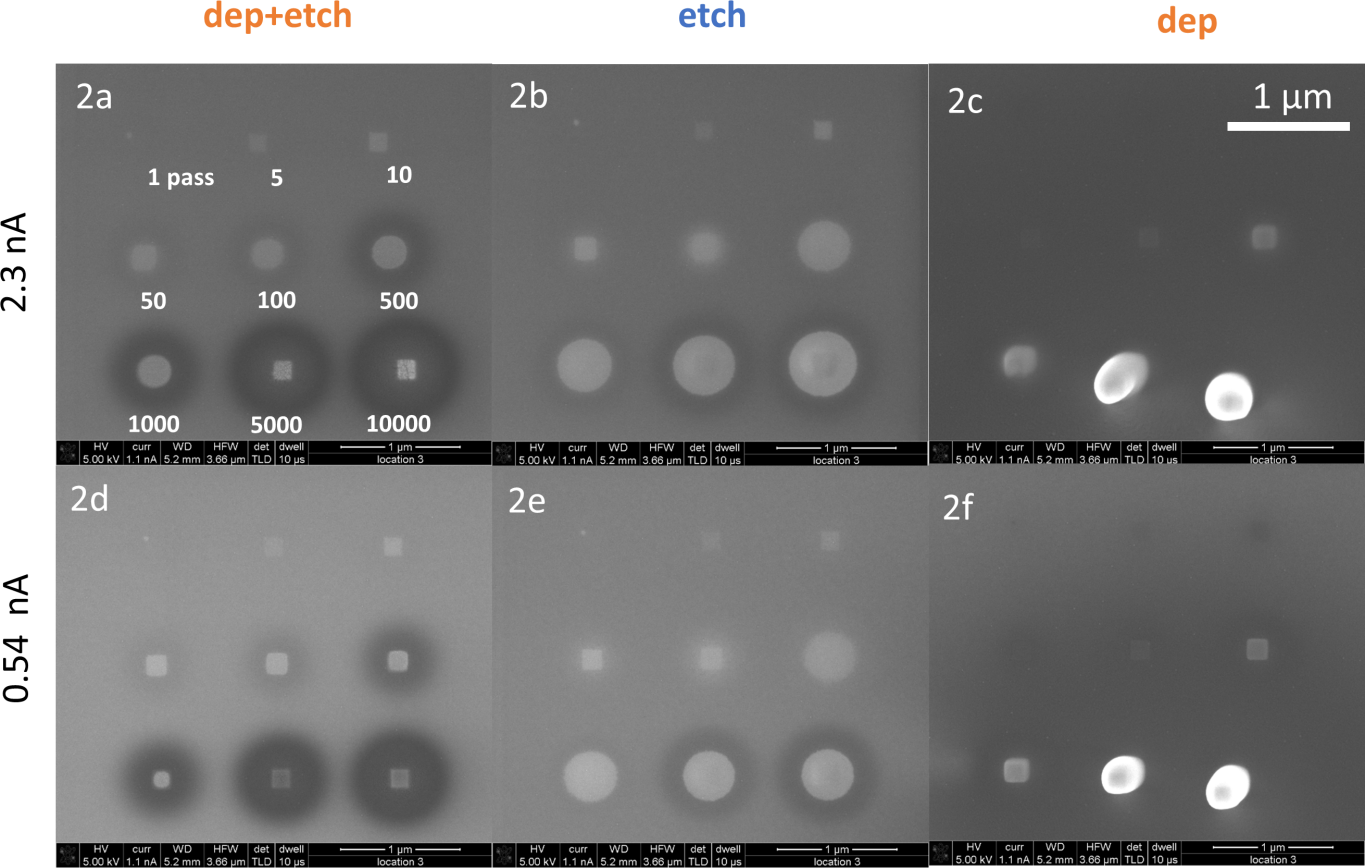
SEM images of square patterns of 150 × 150 nm^2^ (patterned at 5 keV, 10 µs dwell time, 4 nm pitch, 1, 5, 10, 50, 100, 500, 1000, 5000, and 10000 passes, ordered as indicated in the upper left image) at 2.3 nA (**2a**–**2c**) and 0.54 nA (**2d**–**2f**). The right column (**2c**, **2f**) shows deposition only, the middle column (**2b**, **2e**) etching only, and the left column (**2a**, **2d**) simultaneous etching and deposition.

Purification during deposition consists of two simultaneous electron-induced processes, namely, deposition of metal and etching of carbon. The composition of the resulting deposit will depend on the detailed balance between the two processes. More insight in this can be obtained by monitoring the purification for a range of exposure times (number of passes between 1 and 10000) and for two different beam currents of 0.54 and 2.30 nA. In Figure 8, exposure time series are shown for three different situations, that is, one where only Pt deposition was carried out, one where only etching with water was done, and one where both processes occur simultaneously. As the flux in the deposition-only experiment is quite low, the deposits only become visible at intermediate exposure times (100–500 passes). At higher exposure times, the deposits start broadening when the halo starts to develop; at the highest exposure times, deformation of the deposits is seen, which is ascribed to stage drift.

In the etch-only experiments at lower exposure times, only a brightening of the patterning area is observed, indicating cleaning of the substrate, that is, removal of carbon contaminants. After increasing the number of passes to 100 (2.3 nA) or 500 (0.54 nA), the bright region extends outside of the patterning area, and it evolves into a round shape, indicating further cleaning of the substrate in the BSE range. This is in agreement with the observations made in the experimental series **1b**–**1e** (see Figure 5 and Figure 6), where an increase of current (and hence of available electrons in the BSE range) leads to both deposition and etching, resulting in partially purified material within the BSE range. In this case, with the injection of the water precursor only, cleaning of the silicon substrate is the only process observed. At a larger number of passes (5000–10000), a change in the morphology of the patterned area is seen, suggesting further oxidation of the Si substrate (similar to the observation presented in Figure 7) [57]. At the same time, at the perimeter of the BSE range, the formation of a dark halo is observed where the deposition of carbon from contaminants dominates the water-assisted cleaning of the surface.

When deposition and etching are performed at the same time, brightening occurs up to an exposure time corresponding to 1000 passes (at 2.3 nA), even extending beyond the patterning area. It is hard to conclude whether etching is dominant in the entire bright area, or whether also some deposition of purified material occurs in the patterning area. The dark halo developing at the perimeter of the BSE range is due to the deposition of carbon-containing material from both the Pt precursor and contaminants in the system. At 5000 and 10000 passes (at 2.3 nA), a clear deposit becomes visible, which is actually purified as concluded from the cracks in the deposit. The area around the deposit is now covered in a carbon-containing black layer. This means that, in the patterning area, purified deposition dominates, whereas outside this area unpurified deposition occurs. At the lower current of 0.54 nA, the purification in the patterning area is not as complete as at 2.3 nA. By changing the electron beam current, and hence the electron flux, it is possible to control the regimes in which either deposition or purification is the dominant process. These observations are further substantiated by the BSE images of **2a**, **2c**, **2d**, and **2f** (Supporting Information File 1, p S32).

**Figure 9:**
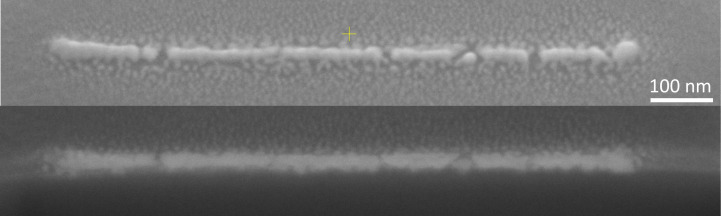
52°-tilted SEM images of **2g** (1 µm line, 5 kV, 0.54 nA, 1 µs dwell, 4 nm pitch, 100000 passes) before FIB milling (top) and after milling (bottom). Imaging and milling were performed in a Thermo Fisher Scientific Helios dual-beam instrument.

The images of the purified structures in Figure 8, sample **2a** at 5000 and 10000 passes, as well as those of the samples **1d** and **1e** in Figure 5, show a granular morphology. To investigate the degree of granularity and to learn whether applications are feasible in which conducting lines are required, line deposits were grown with a length of 1 µm (Figure 9). The finest achieved line at 5 kV and 0.54 nA has both height and width between 30 and 40 nm and mostly consists of connected grains, whereas, around the lines, isolated grains are visible. Also, several gaps are present in the lines, where the grains apparently did not connect to each other. A FIB cross section of the line revealed that the gaps extend down to the substrate surface. The gaps and the granularity may arise from the high mobility of Pt, and especially PtO*_x_* species, on SiO*_x_* substrates, as demonstrated during atomic layer deposition (ALD) experiments, although at elevated temperatures [58–59]. It has been postulated that the growth of Pt nanoparticles through ALD (using MeCpPtMe_3_ as the precursor molecule) is determined rather by the adsorption of migrating Pt species and particle coalescence than by the adsorption of precursor molecules. Furthermore, PtC*_x_* FEBID deposits exposed to e-beam curing showed an increase of Pt granule size, also indicating Pt mobility [60]. The presence of a multilayer of water on the substrate during deposition is a factor that should be considered when comparing the obtained data with other studies of Pt nanoparticle mobility on surfaces.

While the lines obtained at circa 0.5 nA approach a closed structure, lines deposited at higher currents show more substantial cracking and lack of continuity. This limitation is connected to the inherent mobility of Pt and PtO*_x_* species on SiO*_x_* surfaces and could be circumvented by the use of a different substrate.

## Conclusion

Water-assisted purification during FEBID of gold and platinum deposits is achievable under experimental conditions compatible with a standard electron microscope. The purification process highly depends on the ratio of the deposition and etching precursor fluxes and, hence, requires the use of a two-nozzle injection system with enough flexibility to independently control the two precursor fluxes. An always present third flux of contaminants was also found to play a role in the deposition and purification process. Partial purification of gold deposits from the precursor Au(acac)Me_2_ indicates the applicability of the purification protocol reported by Shawrav et al. to a wider range of Au precursors. A maximum purity of 52 atom % gold with a C/Au atom % ratio of 0.5 was achieved. Total purification of the deposit was not achieved because of the water pressure limitations posed by the use of a standard SEM. The possibility of achieving higher chamber pressures, for example, through the use of an environmental SEM (ESEM), would probably allow for the deposition of pure metal. For platinum deposition, the purification of the deposits is more efficient at high electron currents, with the unwanted formation of co-deposited material in the form of a partially purified halo. Through water-assisted purification, the C/Pt atom % ratio has been effectively lowered from 6–7 to 0.2–0.3. While deposition of Pt at high currents leads to high purity, unwanted growth occurs in the proximity of the patterned deposits. A compromise between purity and definition of the deposit shape can be found at low currents (0.13 nA), where the produced material is comprised of partially purified platinum (C/Pt ratio of 0.97). An increase of the electron flux by increasing the dwell time at low beam currents could result in well-defined high-purity Pt deposits. The use of a two-nozzle injection system could be extended to the purification during deposition from other noble metal-based FEBID precursors.

## Supporting Information

Supporting Information File 1 contains the following sections: EDX quantification of the Pt–C material, Water-assisted purification of gold deposits, Co-injection of water and platinum through the same nozzle, Platinum deposit purification: water pressure variation, Variation of the injection order of water and platinum, Effect of water injection on the composition of pre-grown PtC*_x_* deposits, Platinum deposit purification: current variation, SEM-EDX and TEM-EDX comparison of a purified platinum deposit, and Platinum deposit purification: process investigation.

File 1 Additional experimental data.

## Data Availability

All data that supports the findings of this study is available in the published article and/or the supporting information to this article.
