# Supplementary material for: Water-assisted purification during electron beam-induced deposition of platinum and gold
Source: Beilstein J Nanotechnol. 2024 Jul 18;15:884–96. doi: 10.3762/bjnano.15.73 (PMC11285079; doi:10.3762/bjnano.15.73)
Supplement: File 1 — Additional experimental data. [file Beilstein_J_Nanotechnol-15-884-s001.pdf]

BEILSTEIN JOURNAL OF NANOTECHNOLOGY

Supporting Information

for

Water-assisted purification during electron beam-induced deposition of platinum and gold

Cristiano Glessi, Fabian A. Polman and Cornelis W. Hagen

Beilstein J. Nanotechnol. **2024,** *15,* 884–896. doi:10.3762/bjnano.15.73

Additional experimental data

License and Terms: This is a supporting information file under the terms of the Creative Commons Attribution License (https://creativecommons.org/licenses/by/4.0). Please note that the reuse, redistribution and reproduction in particular requires that the author(s) and source are credited and that individual graphics may be subject to special legal provisions.
The license is subject to the *Beilstein Journal of Nanotechnology* terms and conditions: (https://www.beilstein-journals.org/bjnano/terms)

Contents

EDX quantification of the Pt–C material S3

Water-assisted purification of gold deposits S6

Co-injection of water and platinum through the same nozzle S8

Platinum deposit purification: water pressure variation S11

Variation of the injection order of water and platinum S14

Effect of water injection on composition of pre-grown PtC_x deposits S16

Platinum deposit purification: current variation S18

SEM-EDX and TEM-EDX comparison of a purified platinum deposit S29

Platinum deposit purification: process investigation S32

References S33

EDX quantification of the Pt–C material

As mentioned in the Experimental section of the main manuscript, EDX quantification of platinum in the presence of carbon can be tricky because of the overlap of Pt N and C K peaks. In order to clarify this situation, we analysed a sample of pure platinum as a reference material.

A 32 nm thick layer of pure platinum was deposited, by e-beam evaporation with a Temescal FC-20349 evaporator, on top of a Si sample with a 300 nm layer of silicon oxide. A map EDX of the Pt layer was used as a reference for the material. Because platinum forms a homogeneous layer, a map EDX is a better choice to obtain a more reliable signal. In Table S1, a list of EDX peaks as exported from the Oxford Aztec software is presented after performing map EDX of the layer at 5kV and 1.1 nA. The red entries in Table S1 are recognized as the most prevalent EDX signal for each element under the chosen conditions; a distribution around those values was used for the further composition quantification (Table S2). All other entries are known secondary EDX signals which are not quantified by the software.

Table S1: Peak list exported from the Oxford Aztec software for the map EDX of the Pt layer.

keV	element
0.525	O
9.442	Pt
11.071	Pt
11.251	Pt
9.975	Pt
12.942	Pt
13.271	Pt
8.268	Pt
2.05	Pt
2.331	Pt
1.592	Pt
0.258	Pt
0.244	Pt
1.74	Si
0.277	C

Table S2: Composition results of the EDX analysis of the Pt layer, as exported from the Oxford Aztec software.

Map Sum Spectrum	Line Type	Area (cts)	Sigma	Fit Index	Apparent Concentration	k Ratio	Wt.%	Atom %
C	K series	9147.4	323	116.5	2.88	0.02885	3.29	13.5
O	K series	23479.6	414.5	39.4	18.75	0.06309	6.05	18.6
Si	K series	32651.9	478.4	6.2	20.12	0.15946	9.53	16.7
Pt	M series	212253.3	1314	4.5	190.87	190873	202.53	51.1

As can be observed in Table S1, two Pt peaks (244 and 258 eV), unquantified by the software, exist in close proximity to the C K peak (277 eV). These are Pt N peaks, which are erroneously partly included in the quantification of carbon by EDX analysis in PtC_x materials. Mehendale et al. reported on this

effect [1] and found for pure bulk platinum a peak intensity ratio Pt N/Pt M of 0.09. Geier et al. [2] similarly reported a ratio of 0.08 for a 200 nm layer of pure platinum (after background subtraction). The range selected for the overlapping Pt N and C K peaks was 120–330 eV, while the Pt M peak range was 1950–2220 eV.

In this work, the Aztec Oxford software identifies the distribution of counts around 277 eV as the C K peak. However, this distribution also contains counts of the closely lying Pt N peaks, leading to an overestimation of the carbon content (Figure S1). Because of the lack of quantitative information about the Pt N peaks, it is hard to deconvolve the two peaks. The distribution of counts that the Oxford Aztec software generates around the C K value is shown in Figure S1 as the blue line. It results from fitting the count distribution to the peak in the EDX spectrum using a Filtered Least Squares approach, subtraction of background, and correction for overlapping peaks. This can be done for each peak in the spectrum, in particular for C, O, Si, and Pt. The integration of counts under those peaks (displayed as Area in Table S1) allows for the calculation of the peak intensity ratio of C K/Pt M, where C K still contains some of the Pt N counts.

Different routes can be taken in the selection of integration boundaries and in the consideration of the background. We considered two different integration boundaries: the ones utilized by the Oxford software and the ones reported by Geier et al. While the effect of the C K/Pt M range on the selection of this integration range seems severe, this effect diminishes for bigger peaks.

Furthermore, the background can be subtracted or corrected. As such, we consider two background corrections: the correction performed by the Oxford software and a background correction (Table S3) performed by roughly subtracting the background signal (for the 120–330 eV range, a linear background from 0 to 340 eV, for 1950–2220 eV a linear background from 1940 to 2230 eV).

No clear background subtraction is possible: Bremsstrahlung X-rays at any given energy could be calculated from Kramer's law, but a large amount of low-energy Bremsstrahlung X-rays are adsorbed in the sample or detector. Additionally, the generation of Bremsstrahlung X-rays at any given energy is dependent on the mean atomic number, meaning that the calculation of a background spectrum without Pt is not reliable.

Background subtraction (Table S3) to mimic Geier's measurements is performed by subtracting a spectrum of silicon with native silicon oxide from the spectrum of the reference Pt sample. Because of the high sensitivity of our detector to low-energy X-rays, we record also the Si Lα signal at 0.09 eV, which is subtracted from the reference spectrum between 0.12 and 0.16 eV.

For the 32 nm Pt layer (reference material) the C K/Pt M ratio using the Oxford method was found to be 0.04. This value is lower than the previously reported values, which is due to the partial removal of the Pt N peak. If a similar method of the one reported by Geier et al. is applied to the same sample, the ratio would be increased to 0.11, in line with previous reports. As such, a count ratio of 0.04 between the C K and Pt M peaks is assumed to be pure platinum. The count ratio can be directly related to the calculation of the weight % ratio and then atomic % ratio. As such an atomic % C/Pt ratio of 0.26 corresponds to pure Pt. A more complete overview of possible ratios obtained with different quantification methods and using different peak ranges is presented in table S3.

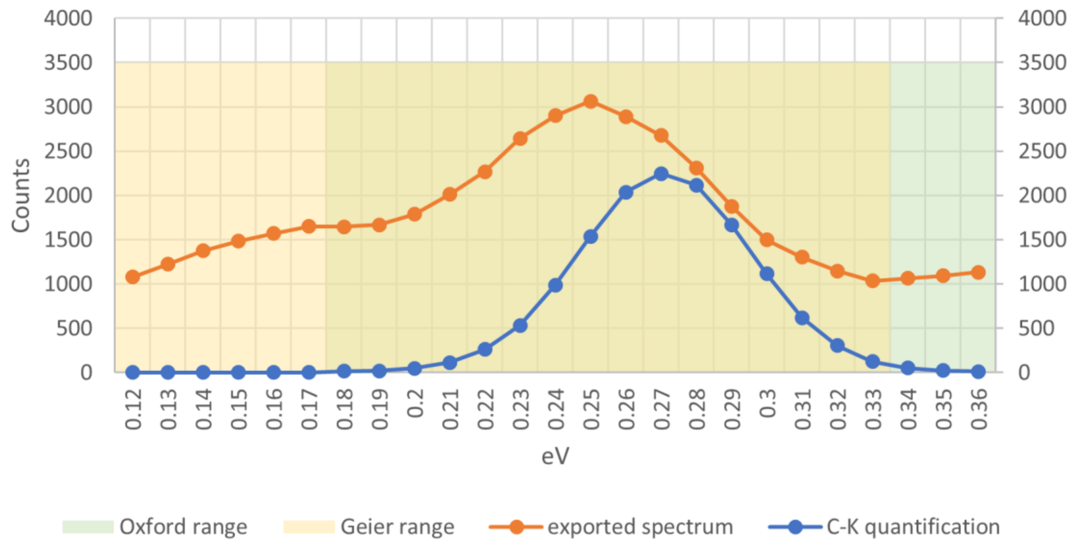

Figure S1: Exported spectrum and C K peak quantification for the EDX spectrum of the Pt layer exported from the oxford Aztec software. Graphical representation of the ranges used for the C K quantification by the Oxford software (0.18–0.36 keV) and by Geier et al. (0.12–0.33 keV).

Table S3: Comparison of several peak integration methods for the Pt and C EDX signals.

	Pt-N, C-K (counts), 120-330 eV	Pt-N, C-K (counts) 180-360 eV	Pt-M (counts) 1950-2220 eV	Pt-M (counts)	ratio
Oxford integration		9147.4		212253.3	0.04
no background correction	41122		205616		0.20
no background correction		36027	205616		0.18
background correction	24815.5		172554.5		0.14
background correction		19332	172554.5		0.11
background subtraction	21564		189950.3		0.11
background subtraction		20586.05	189950.3		0.11

The parameters for point EDX were: acquisition time 30 s and pulse pile-up correction. Data are presented in atomic percentage (atom %), all elements are selected manually, as the C and O peaks are sometimes not auto-picked. For map EDX: 50 frame count, resolution 256, pixel dwell time 100 µs, and frame live time 6 s. For line EDX: 50 passes, pixel dwell time 5 ms, number of points 500, and pass live time 3 s. Lines are in atom %, presented with Si included, unless specified otherwise. All horizontal lines are from left to right. Data were exported with three-point averaging. A quantification error of ±2 atom % can be expected.

Water-assisted purification of gold deposits

This section contains the supporting information for the water-assisted purification of gold deposits. The values plotted in Figure 2 of the main manuscript are listed in Table S4, together with the O and Si content. The complete plot of atom % composition versus chamber pressure increase for all detected elements (C, O, Si, and Au) and is shown in Figure S2. The positioning of the GIS needles during the gold deposition experiments is depicted in Figure S3. The patterning conditions were: 200 µs dwell time, 10 keV, 2 nA, 4 nm pitch, and 100 passes.

Table S4: Elemental analysis, from EDX measurements, of gold deposits obtained at different SEM chamber pressure increments after precursor injection. The atomic percentages of C, O, Si, and Au are presented here and plotted in Figure S2. The C/Au atomic percentage ratio is presented in the last column. These are the values plotted in Figure 2 of the main manuscript.

Pressure increase after precursor injection (10^{-5} mbar)	C (atom %)	O (atom %)	Si (atom %)	Au (atom %)	C/Au
0.053	55.7	9.52	8.98	25.8	2.16
0.079	56.4	9.62	8.78	25.2	2.24
0.136	55.9	9.5	9.4	25.2	2.22
0.205	56.6	9.56	8.64	25.2	2.25
0.305	54.5	9.63	9.67	26.2	2.08
0.384	54.7	10.1	9.3	25.9	2.11
0.7	54.8	10.1	9.1	26	2.11
0.916	51.5	8.5	8.5	31.5	1.63
0.951	45.7	7.8	10.8	35.7	1.28
1.1	53.8	10.2	9.6	26.4	2.04
1.38	52.2	9.29	9.21	29.3	1.78
1.62	48.2	9.6	10.5	31.7	1.52
2.14	53.4	5.12	7.88	33.6	1.59
2.32	41.5	6.4	10.8	41.3	1.00
2.36	33.4	5.6	13.4	47.6	0.70
2.9	33	5	13.4	48.6	0.68
3.31	27.9	4.2	14.5	53.4	0.52
3.46	33	5.1	14	47.9	0.69
3.46	28.9	4.7	14.5	51.9	0.56
4.01	25.3	4.2	17.9	52.6	0.48

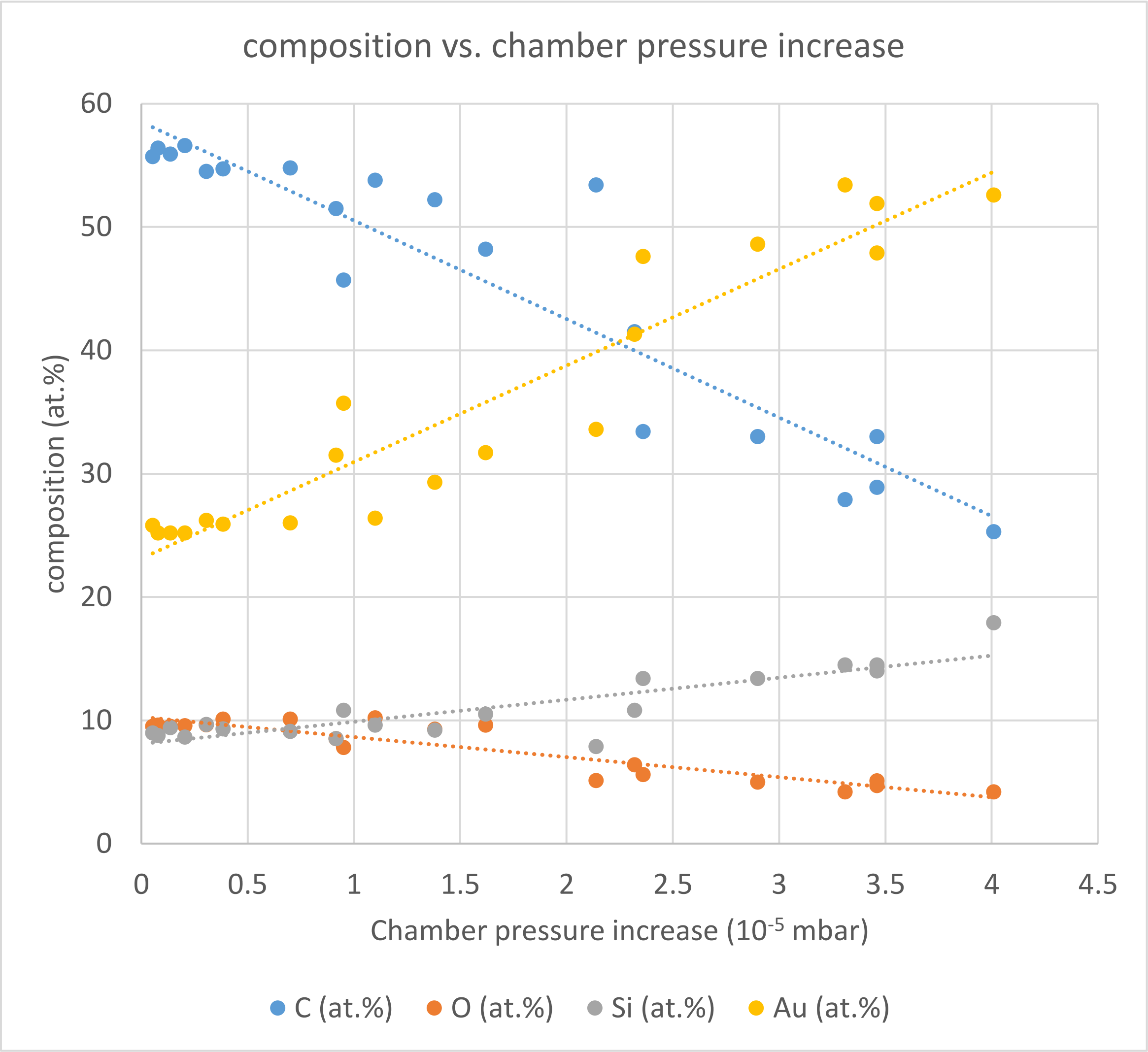

Figure S2: A complete plot of the composition of deposits in atom % of Au, C, O, and Si for gold deposition experiments as a function of the chamber pressure increase during deposition. Data are fitted with linear trend lines as a guide to the eye.

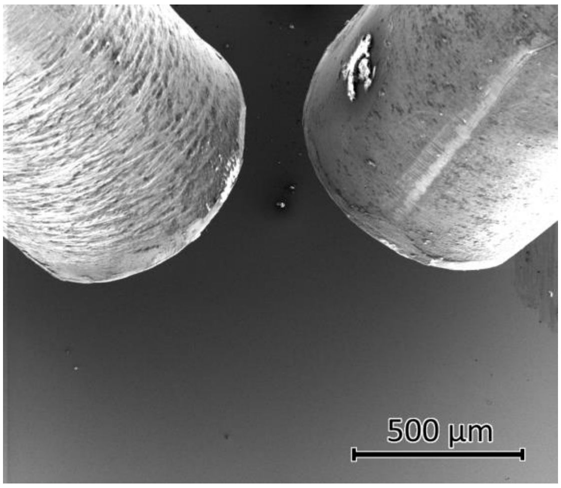

Figure S3. Gold (left) and water (right) GIS needle positions for gold purification experiments.

Co-injection of water and platinum through the same nozzle

Co-injection of platinum precursor and water precursor through the same nozzle was tried using the FEI GIS for TEOS deposition, which is equipped with an additional external reservoir. To explore the effect of the injection through the same nozzle of the two precursor, several experiments were performed. First, a deposit was performed with Pt injection only (**3a**), followed by a deposit performed with water injection only (**3b**). Subsequently another deposit with water injection only was performed (**3c**), followed by patterning without precursor injection (**3d**), and, finally, deposition was performed with both platinum and water injection (**3e**) (Figure S4).

The composition of the deposits was analysed by point EDX (Table S5). For the injection of platinum only, a C/Pt ratio of 3 was obtained. The same C/Pt ratio was observed also for the co-injection of Pt and water (**3e**) and, surprisingly, also for the first experiment with injection of water only (**3b**). This is most probably due to the water flux, which transports left-over platinum precursor, adsorbed to the inside of the GIS system, to the deposition area. Pt material is likely to get adsorbed (or even condensed) inside of the GIS because of the low temperature of the GIS during the experiments. Surprisingly, for experiment **3c**, performed under the same conditions as **3b**, the deposit obtained presents a much lower C/Pt ratio of 0.3. The much lower amount of available Pt precursor after depositing **3b** led to the deposition of a much thinner, but nevertheless purer, Pt layer. The fact that only in the second water injection deposit a lower C/Pt ratio was observed indicates that a much smaller supply of Pt precursor, in comparison to water precursor, is necessary.

Deposits **3a**–**3e** were grown under the following conditions: Co-injection of platinum and water was performed using a two-precursor FEI GIS, which is normally employed for TEOS deposition. A GIS crucible is loaded with precursor (originally TEOS, in this case $MeCpPtMe_3$) and a modified needle with a side entry is connected. A hose, connected to an external water precursor reservoir, is connected to the side entry of the GIS needle. The water precursor reservoir is separated from the system by a valve, and the flow is controlled through a needle valve. The reservoir and hose are not heated. The system was assembled without backflow limiter on the needle. The needle was positioned roughly 250 µm above the substrate. The Pt reservoir was kept at 30 °C, and the water reservoir was at room temperature (circa 23 °C). The background chamber pressure was $4.68 \cdot 10^{-6}$ mbar. All deposits were done using the following parameters: 2 × 2 μm^2 square deposition area, 200 µs dwell time, 5 kV, 4 nm pitch, and 13 nA of current. All depositions and EDX characterizations for this set of experiments were performed in a TFS Helios dual-beam system. The EDX characterizations were performed with an EDAX Apollo 50 detector, and quantifications were performed with the Genesis software. EDX data from this detector is considered to be qualitative.

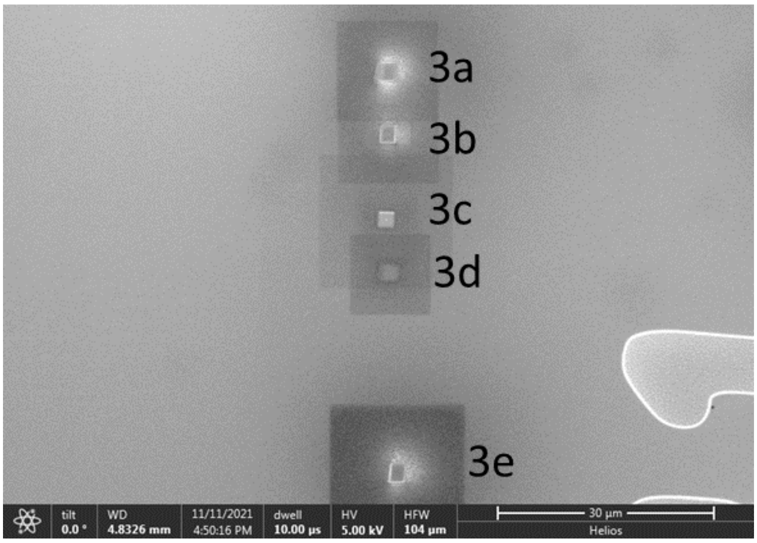

Figure S4: SEM image of the deposits obtained by co-injecting water and platinum through the same nozzle. **3a**–**3e** are ordered from top to bottom in the image.

Table S5: EDX composition results for the deposits **3a**–**3e** produced with a two-precursors GIS. The composition has been calculated without Si because of the high thickness variation of the deposits.

Deposit number	Injected material	C (atom %)	O (atom %)	Pt (atom %)	C/Pt ratio	Pressure during deposition (10^{-5} mbar)
**3a**	Pt only	72	4	24	3	0.681
**3b**	Water only	63	17	20	3.2	6.67
**3c**	Water only repeat[a]	16	36	49	0.3	5.37
**3d**	No injection[a]	66	14	20	3.3	0.583
**3e**	Pt + water	67	6	27	2.5	6.12

[a]Spectrum estimation is not reliable because of the large Si peak.

To better characterize what happens between the depositions **3b** and **3c**, a series of nine deposits was patterned, one directly after another. Deposits **3f**–**3n** were deposited after injection of platinum and with only water injection during the deposition. The resulting deposits show a decrease of deposited material (Figure S5) together with a decrease of the C/Pt atom % ratio (Table S6). This confirms the observations of the previous experiments that the variation of supply of Pt precursor (adsorbed inside of the GIS needle) and water precursor is fundamental in the purification process of the deposit.

Deposits **3f**–**3n** were grown under the following conditions: The double GIS was inserted, and the Pt portion of the GIS was opened for 8 min. After that time, the Pt portion of the GIS was closed. After one minute, the water reservoir was opened, and patterning was immediately started. Nine circular areas were patterned in series (**3f**–**3n**, Figure S5, Table S6). Each circular area was 200 nm in diameter and was deposited at 5 kV, 3.2 nA, 400 passes, and 200 µs dwell time. The patterned areas were separated by 3 µm. The background chamber pressure was $4.35 \cdot 10^{-6}$ mbar, and upon injection of Pt the pressure rose to $5.61 \cdot 10^{-6}$ mbar. During water injection the chamber pressure was $6.88 \cdot 10^{-5}$ mbar.

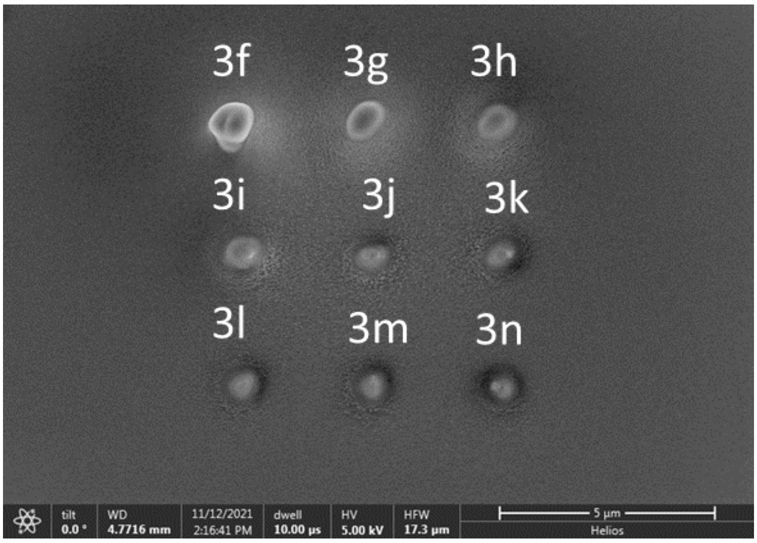

Figure S5: SEM image of the circular deposits **3f**–**3n** deposited after injection of Pt precursor (before patterning) and during injection of water precursor (during patterning).

Table S6: EDX composition results for the circular deposits produced with a two-precursors FEI GIS (**3f**–**3n**). The composition has been calculated without Si because of the high thickness variation of the deposits.

Circle	Start time (after Pt flow stop) (s)	C (atom %)	O (atom %)	Pt (atom %)	C/Pt
**3f**	60	66	10	24	2.8
**3g**	224	63	14	23	2.7
**3h**	388	49	15	36	1.4
**3i**	552	41	17	42	1.0
**3j**	716	33	32	35	0.9
**3k**	880	35	28	37	1.0
**3l**	1044	27	29	44	0.6
**3m**	1208	22	38	40	0.6
**3n**	1372	24	28	48	0.5

Platinum deposit purification: water pressure variation

The EDX spectra from which the C/Pt atom % ratios presented in Figure 3 of the main manuscript were determined, are presented here in Figures S6–S10. The patterning conditions were: 250 × 250 nm^2, 1 µs dwell time, 5 keV, 2.3 nA, 4 nm pitch, and 150000 passes. The background pressure was $9.19 \cdot 10^{-7}$ mbar.

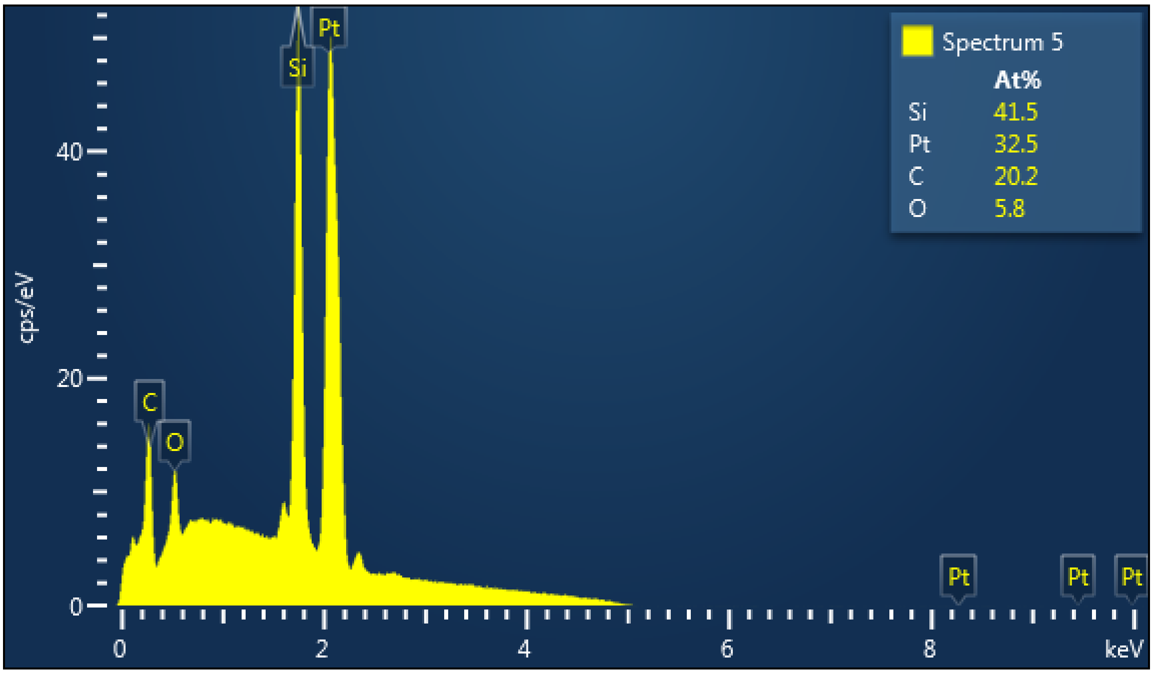

Figure S6: EDX spectrum of the Pt deposit grown at $4.83 \cdot 10^{-5}$ mbar (injection of platinum and water).

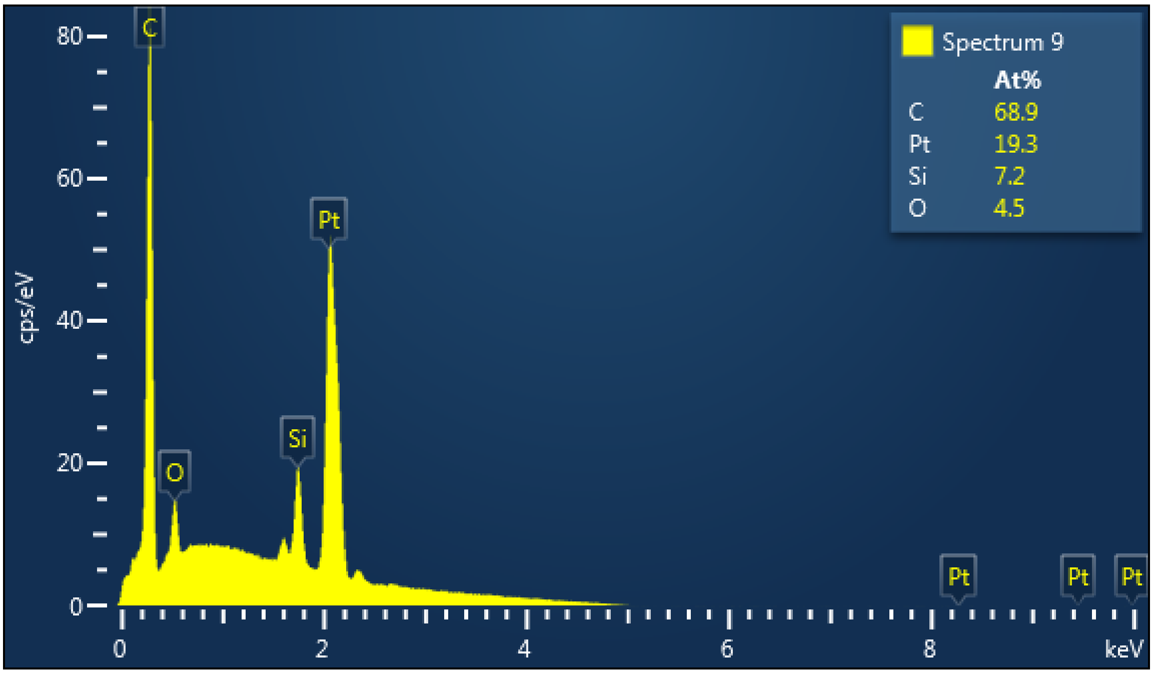

Figure S7: EDX spectrum of the Pt deposit grown at $3.58 \cdot 10^{-5}$ mbar (injection of platinum and water).

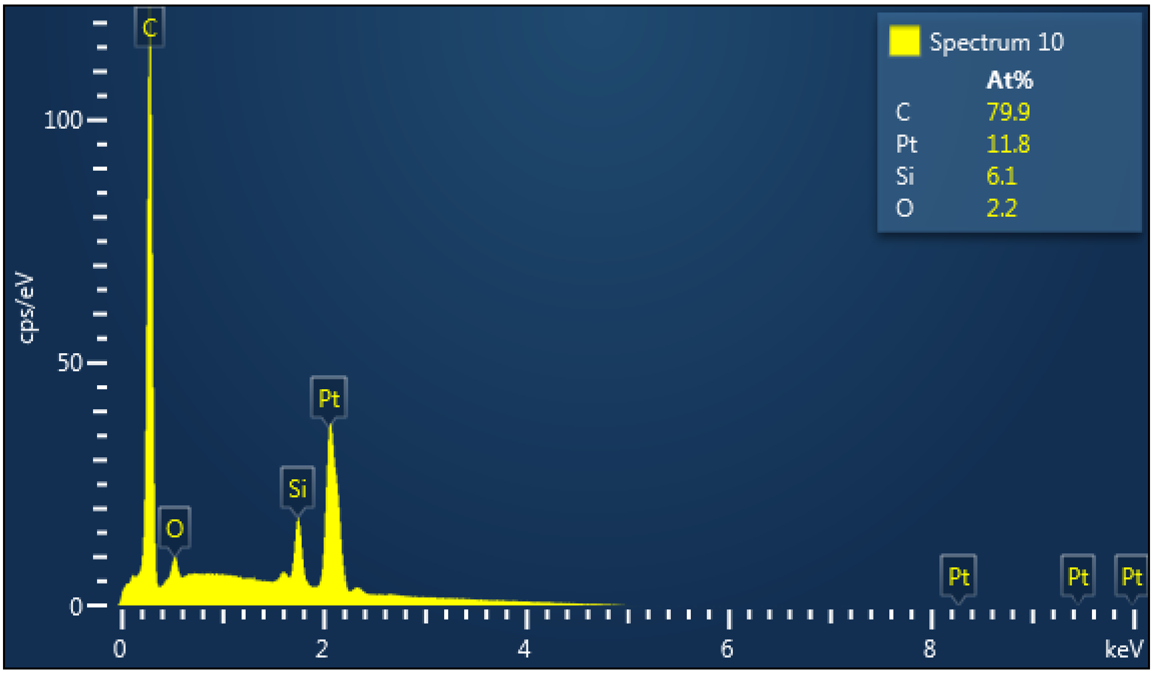

Figure S8: EDX spectrum of the Pt deposit grown at $2.34 \cdot 10^{-5}$ mbar (injection of platinum and water).

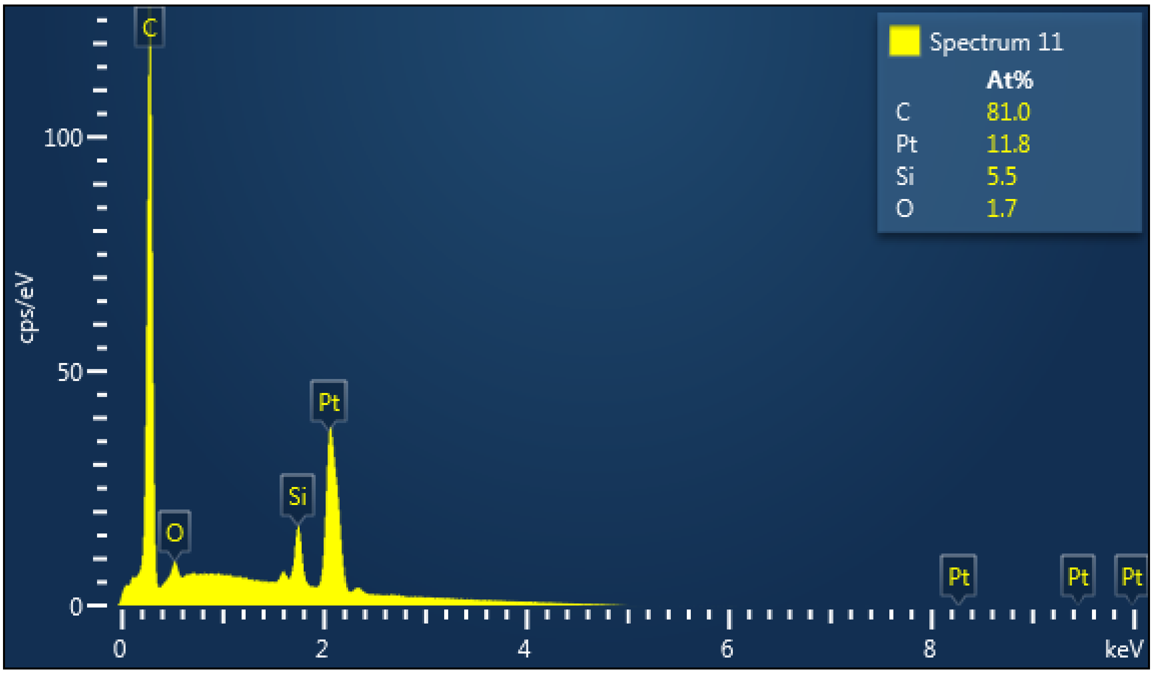

Figure S9: EDX spectrum of the Pt deposit grown at $1.40 \cdot 10^{-5}$ mbar (injection of platinum and water).

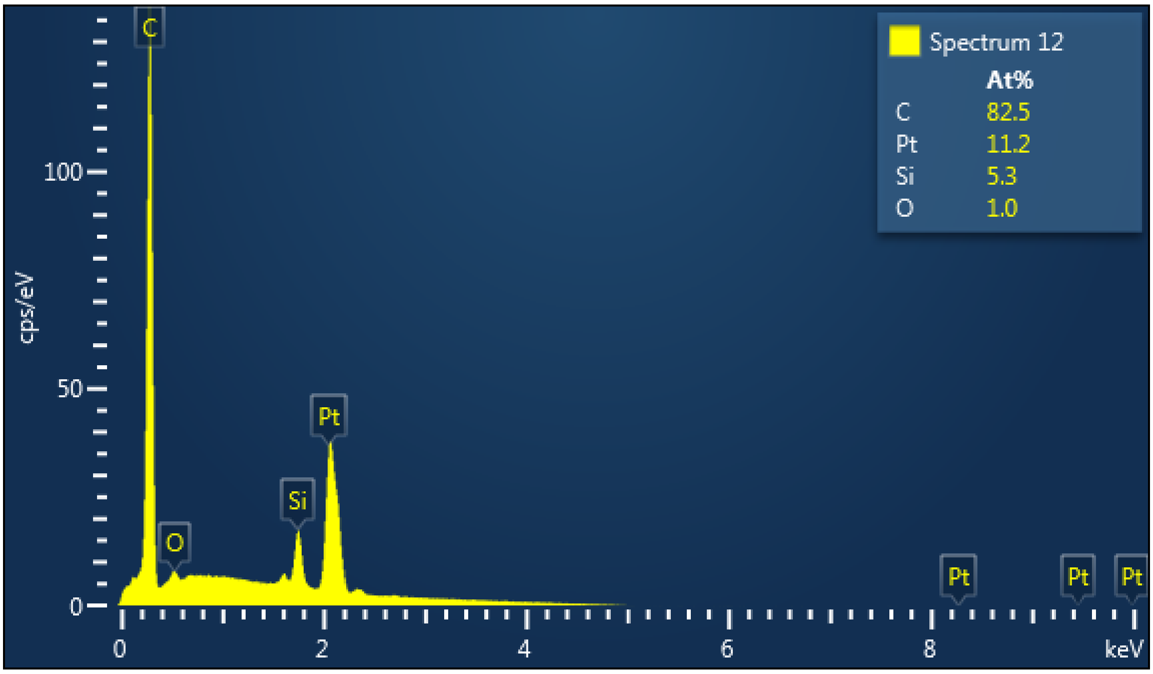

Figure S10: EDX spectrum of the Pt deposit grown at $9.19 \cdot 10^{-7}$ mbar (injection of platinum).

Variation of the injection order of water and platinum

In order to test if the order of opening of the Pt GIS and water GIS has an effect on the deposition composition, the order of opening of the reservoirs was reversed from water-open first (**3o**) to platinum-open first (**3p**) while keeping all other experimental parameters constant (Figure S11, Table S7). No appreciable difference in morphology and composition (obtained by EDX analysis) between the two deposits was observed.

A second test was performed in order to test if the growth of the platinum deposit is a dynamic process with continuous deposition and, hence, dynamic supply of platinum precursor. Because of the remote position of the Pt GIS and the low obtained growth rate of purified deposits, the deposition of pure Pt material could be caused only by adsorbed Pt precursor at the beginning of the deposition experiment. Depositions were performed for different total times to test if the process is dynamic or not. The direct comparison between the EDX composition of **3p** and the shorter experiment **3q** (Figure S11), which are produced under the same experimental conditions, but with **3q** having on fifth of the deposition time of **3p** (Table S7), shows an increase in the EDX spectra of the Pt peak compared to the background Si, indicating a thicker deposited layer for longer deposition times (**3p**).

Deposits **3o**, **3p**, and **3q** were grown under the following conditions: Pt injection, 250 × 250 nm^2, 1 µs dwell, 5 kV, 4 nm pitch, 4.84 nA, and 100000 passes (**3o** and **3p**) or 20000 passes (**3q**), background chamber pressure (1.64–1.68)·10^{-6} mbar, chamber pressure during deposition (4.25–4.96)·10^{-5} mbar. **3o**: H_2O open, stabilization for 2 min, Pt open, stabilization for 2 min, start pattern. **3p**, **3q**: Pt open, stabilization for 2 min, H_2O open, stabilization for 2 min, start pattern.

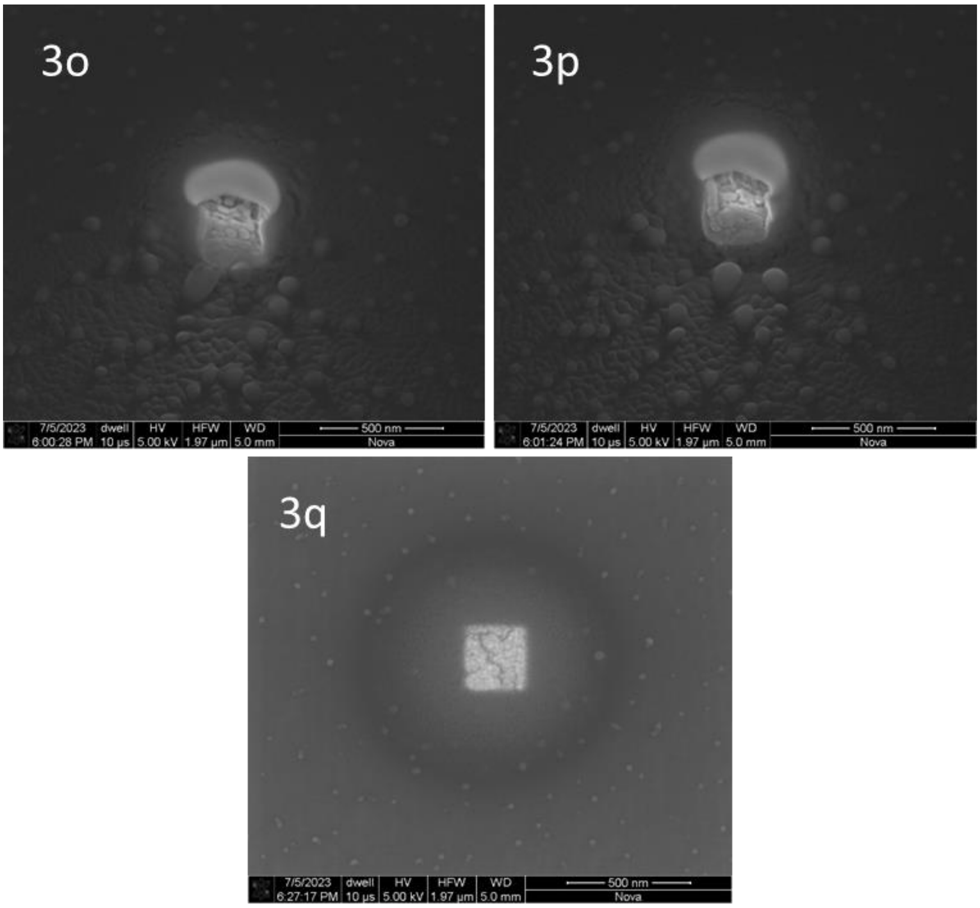

Figure S11: SEM images of deposits **3o**, **3p**, and **3q**.

Table S7: EDX composition table of deposits **3o**, **3p**, and **3q**.

Deposit	C (atom %)	O (atom %)	Si (atom %)	Pt (atom %)	C/Pt
**3o**	17.4	16	12.7	53.9	0.32
**3p**	14.8	9.7	12.1	63.3	0.23
**3q**	4.5	9.3	79	7.2	0.63

Effect of water injection on composition of pre-grown PtC_x deposits

Consecutive deposition and purification of platinum deposits in close proximity to each other could lead to cross-contamination. To test this possibility, three deposits were grown by injection of platinum only under identical conditions and in proximity to each other (**3r**–**3t**, Figure S12). Subsequently, **3t** was patterned again (**3u**) using in this case only water as precursor, effectively performing post-deposition water-induced purification (Figure S13). The EDX analysis of the deposits show no variation of composition for **3r** and **3s** before and after water-purification of **3t** (Table S8). Furthermore, even the unpurified area of **3t** (outside of the **3u** pattern) presents no appreciable purification (Table S8, Figure S14). Only the halo region on the left to the patterned area (**3u**) was effectively purified because of the high amount of scattered electrons generated from the patterning of the tall structure **3t**.

Deposits **3r**, **3s**, and **3t** were grown under the following conditions: Pt injection, 250 × 250 nm^2 square deposition area, 1 µs dwell time, 5 kV, 4 nm pitch, 4.84 nA, 100000 passes, background chamber pressure $1.72\cdot10^{-6}$ mbar, chamber pressure during deposition $1.72\cdot10^{-6}$ mbar. **3t** has been additionally exposed to the pattern **3u**: H_2O injection, 200 × 450 nm^2, 1µs dwell, 5 kV, 4 nm pitch, 4.84 nA, 100000 passes, chamber pressure $3.82\cdot10^{-5}$ mbar.

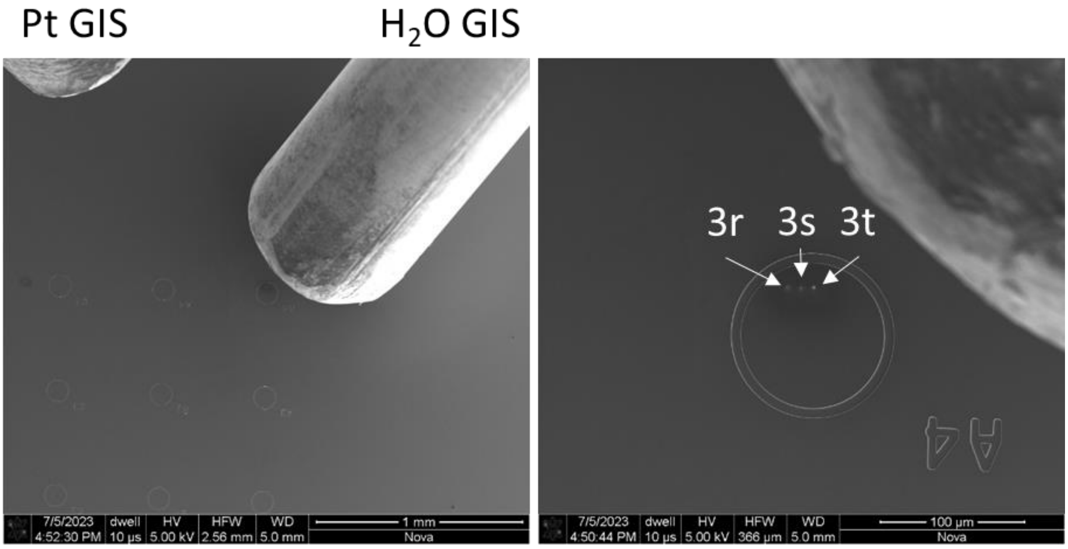

Figure S12: SEM images of the deposition area and relative positioning of the Pt GIS and H_2O GIS (left) and position of deposits **3r**–**3t** under the H_2O needle (right).

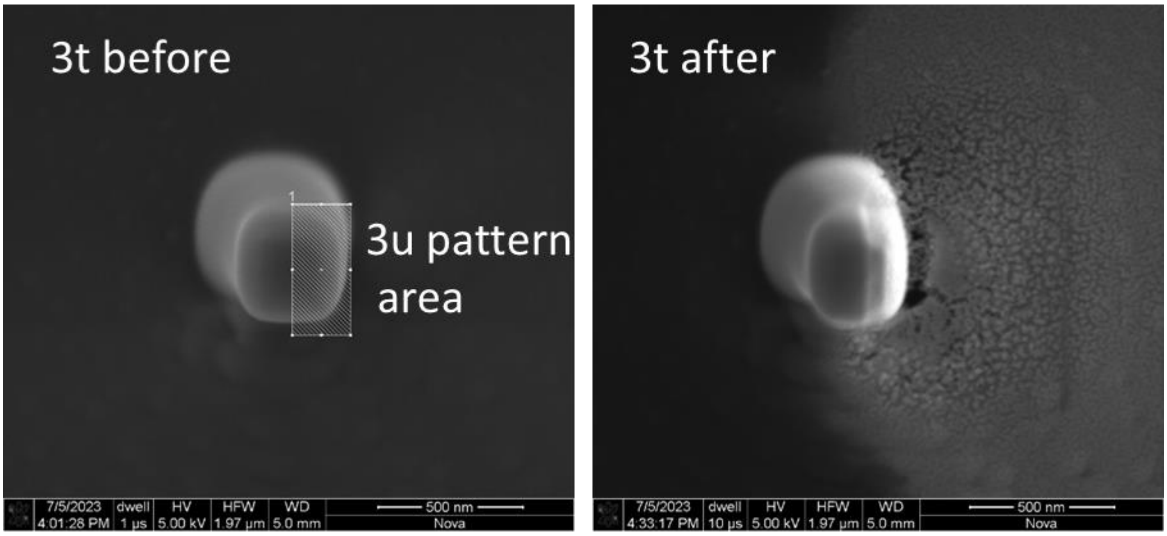

Figure S13: SEM image of **3t** and overlaid pattern area **3u** before (left) and after (right) patterning during water injection.

Table S8: EDX composition table of deposits **3r**, **3s**, and **3t** before and after **3u.**

Deposit	Before/after **3u**	C (atom %)	O (atom %)	Si (atom %)	Pt (atom %)	C/Pt
**3r**	before	79.9	2.1	6.5	11.5	6.9
**3r**	after	81.2	1.7	5.6	11.4	7.1
**3s**	after	81.9	1.5	5.2	11.4	7.2
**3t** (patterned area)	after	48.9	7.9	19.8	23.3	2.10
**3t** (unpatterned area)	after	76.1	2.6	9	12.2	6.2

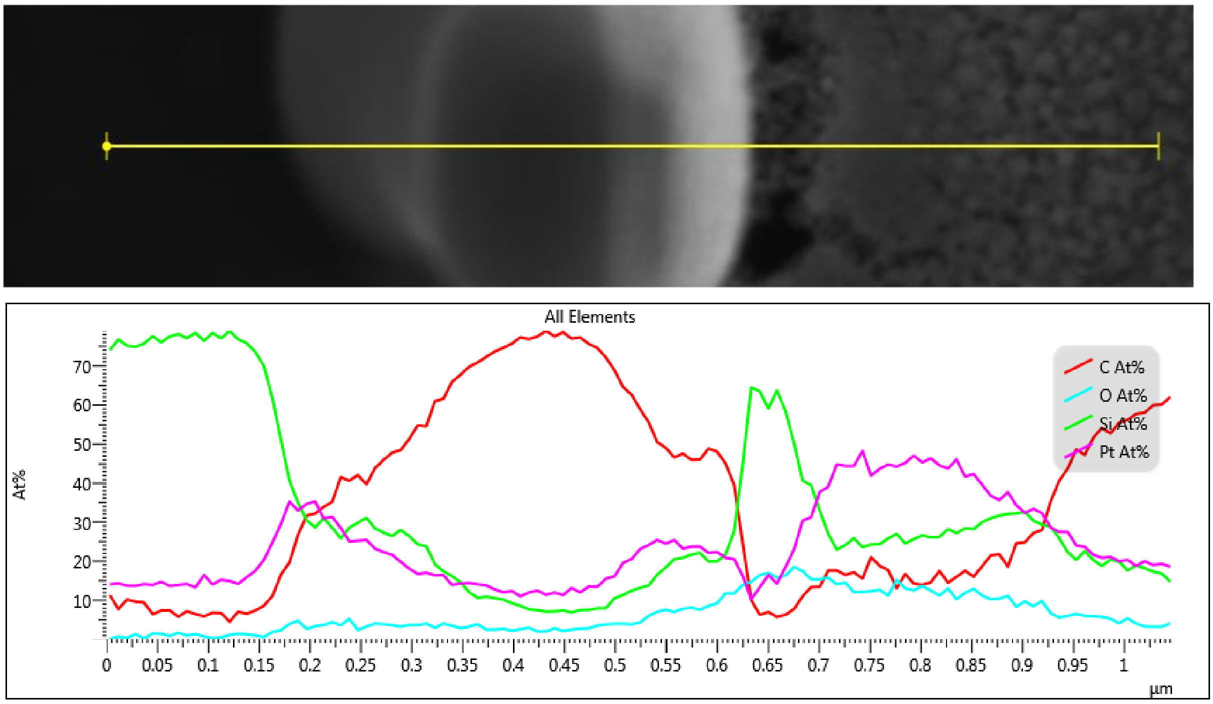

Figure S14: SEM image and composition values of the line scan EDX of **3t** after **3u** patterning.

Platinum deposit purification: current variation

The EDX data regarding deposits **1a**–**1f** are presented here fully in Figures S15–S20. For deposits **1b**–**1f**, the following data are presented in this order: point EDX in the centre of the deposit, EDX layered image (SEM image + EDX maps), EDX map for the main peaks of Si, O, C, and Pt. The patterning conditions of platinum deposits with current variation were: **1a:** 400 × 400 nm^2, 1 µs dwell time, 5 keV, 0.538 nA, 4 nm pitch, 400000 passes, chamber pressure during deposition $(4.13–4.89)\cdot10^{-5}$ mbar. The background chamber pressure was $1\cdot10^{-6}$ mbar. **1b**–**1f:** 250 × 250 nm^2, 1 µs dwell time, 5 keV, 4 nm pitch, 150000 passes, 0.125 nA (**1b**), 0.538 nA (**1c**, **1f**), 2.25 nA (**1d**), and 2.69 nA (**1e**). Chamber pressure during deposition: $5.10\cdot10^{-5}$ mbar (**1b**), $4.76\cdot10^{-5}$ mbar (**1c**), $4.89\cdot10^{-5}$ mbar (**1d**), $4.62\cdot10^{-5}$ mbar (**1e**). Background pressure is estimated to be $1\cdot10^{-6}$ mbar.

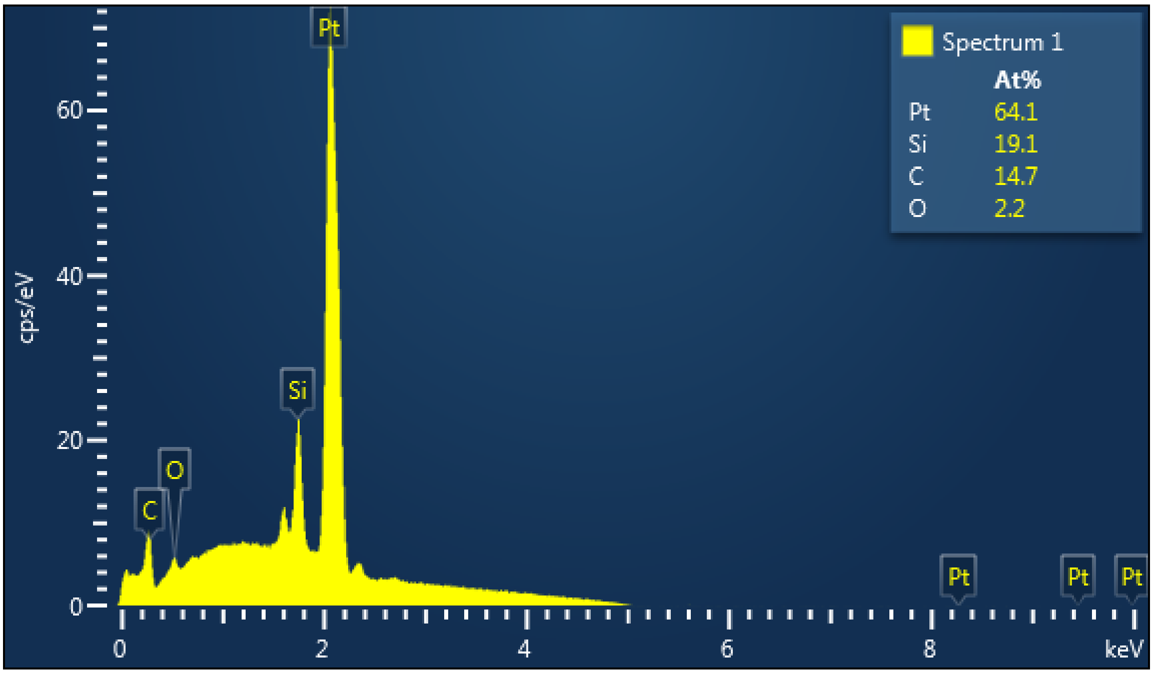

Figure S15: EDX spectrum of Pt deposit **1a**.

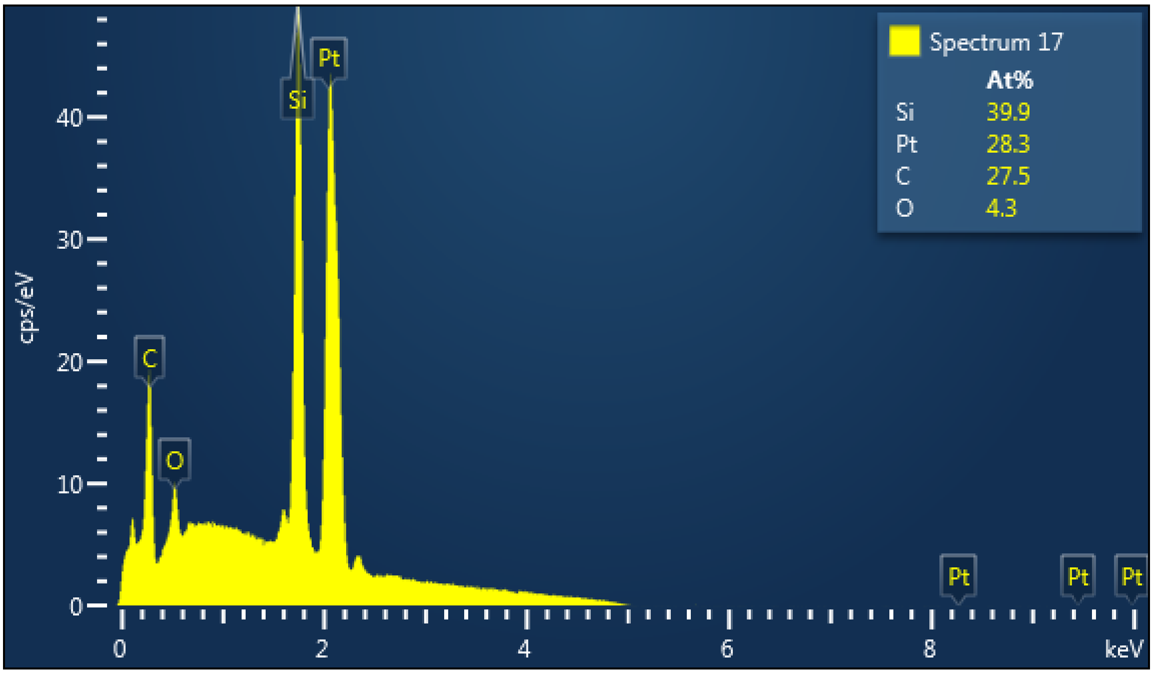
Spectrum 17
At%
Si 39.9
Pt 28.3
C 27.5
O 4.3
cps/eV
keV
0
10
20
30
40
0
2
4
6
8
C
O
Si
Pt
Pt
Pt
Pt

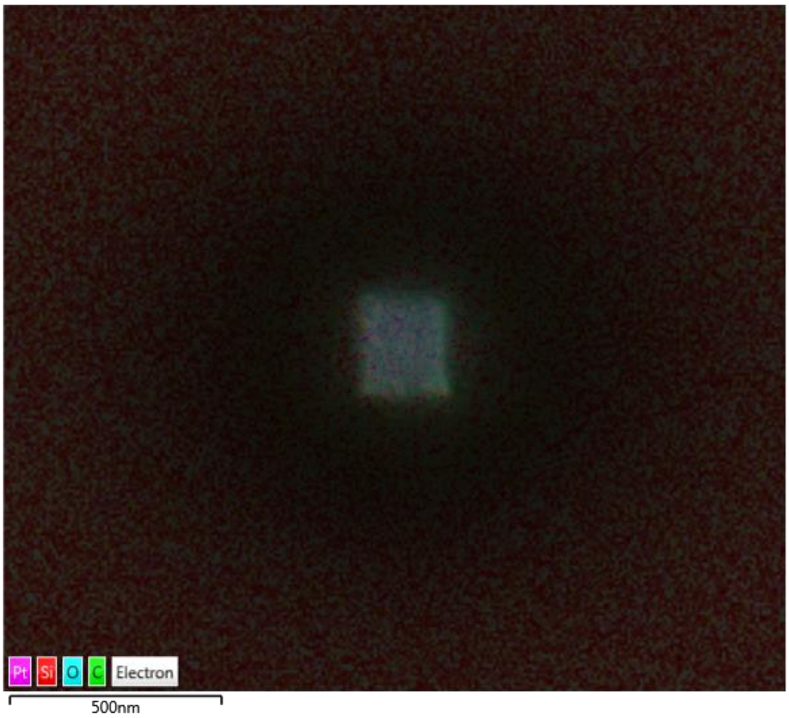
Pt
Si
O
C
Electron
500nm

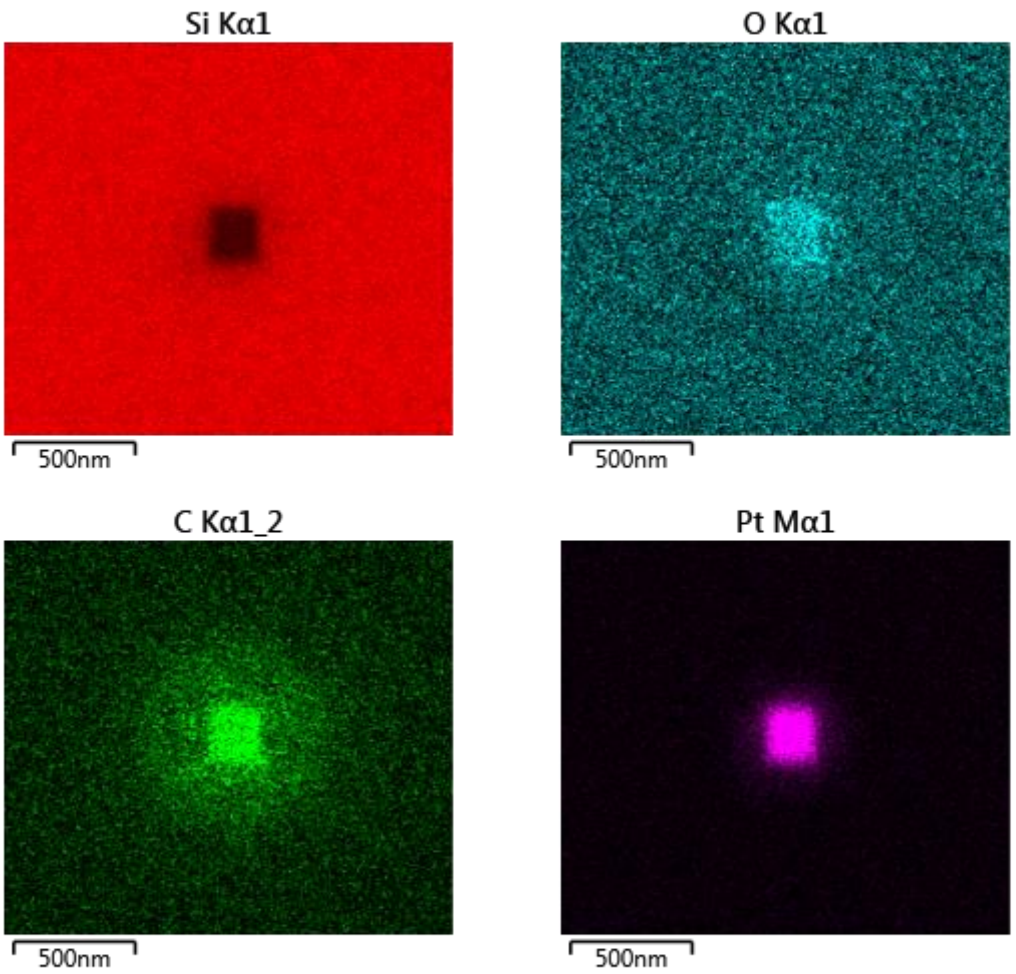

Figure S16: Top: EDX spectrum of Pt deposit **1b**, middle: EDX layered image (SEM image + EDX maps), bottom: separate EDX maps for the detected elements.

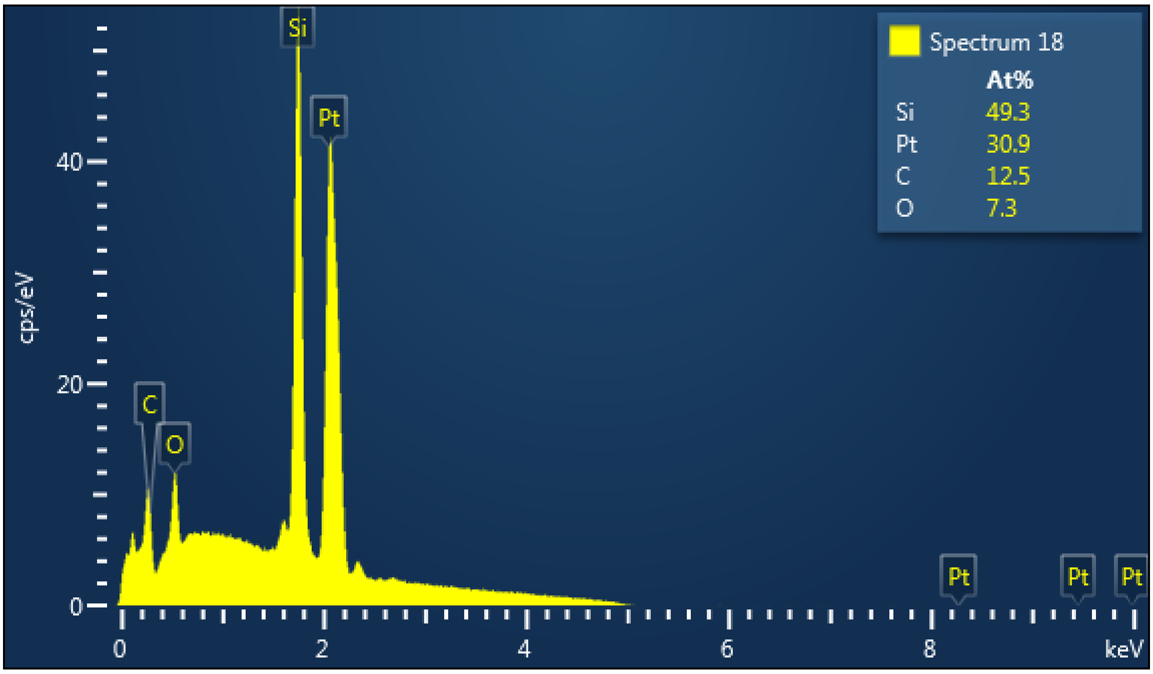
Spectrum 18
At%
Si 49.3
Pt 30.9
C 12.5
O 7.3
cps/eV
0
20
40
0
2
4
6
8
keV
Si
Pt
C
O
Pt
Pt
Pt

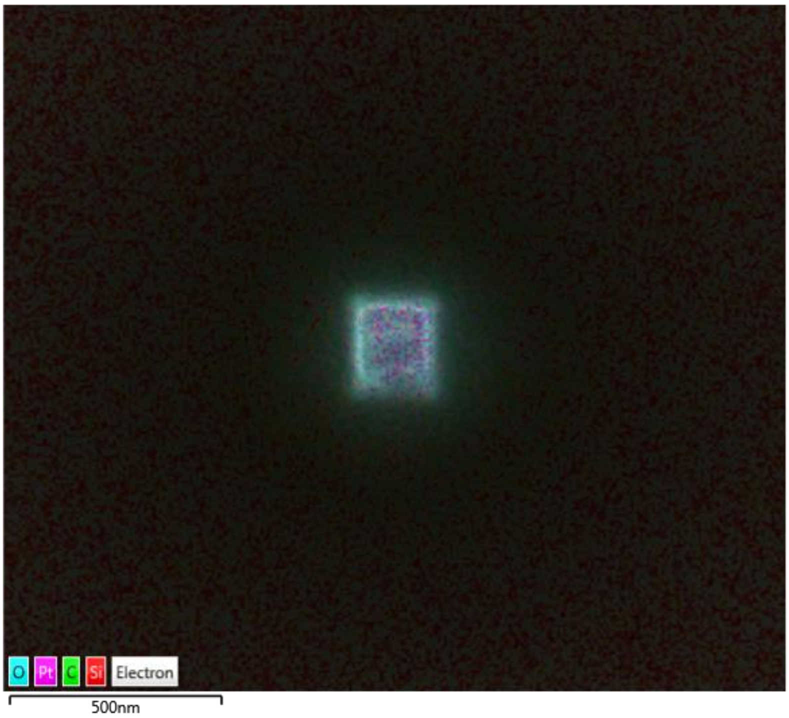
O
Pt
C
Si
Electron
500nm

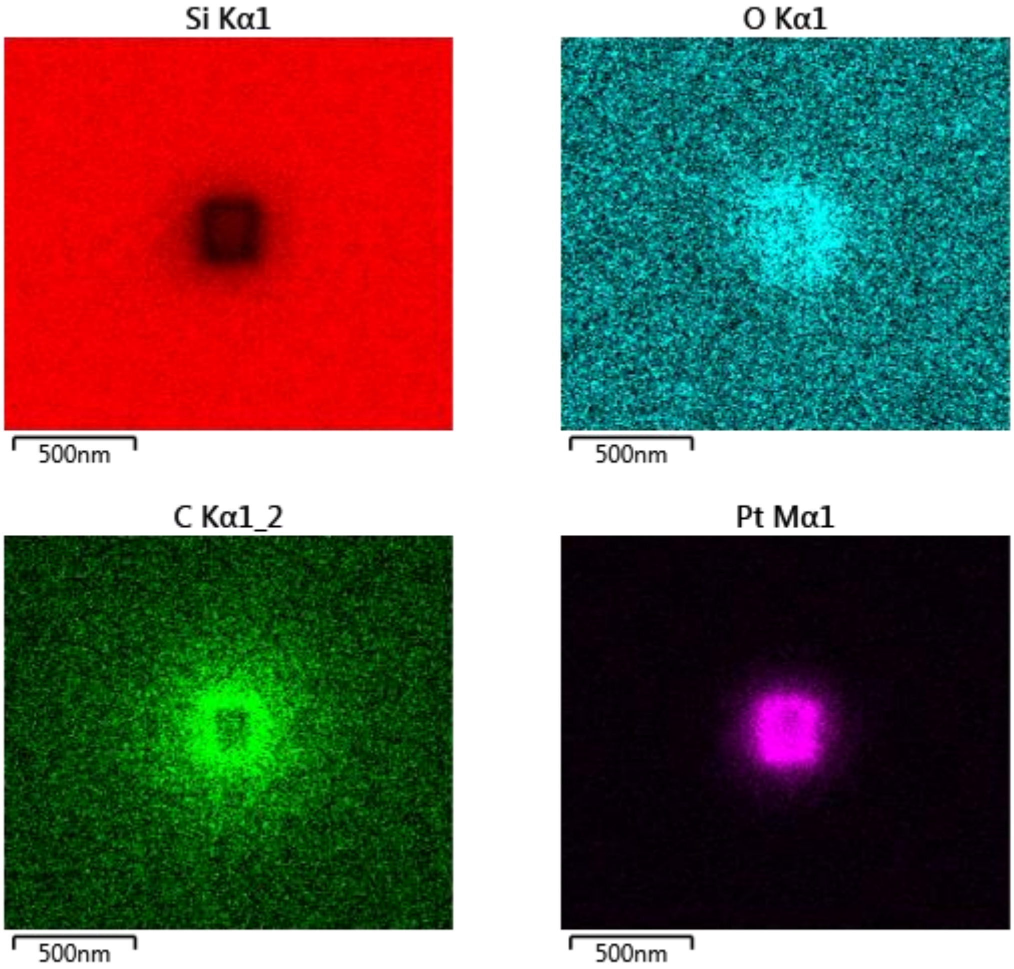

Figure S17: Top: EDX spectrum of Pt deposit **1c**, middle: EDX layered image (SEM image + EDX maps), bottom: separate EDX maps for the detected elements.

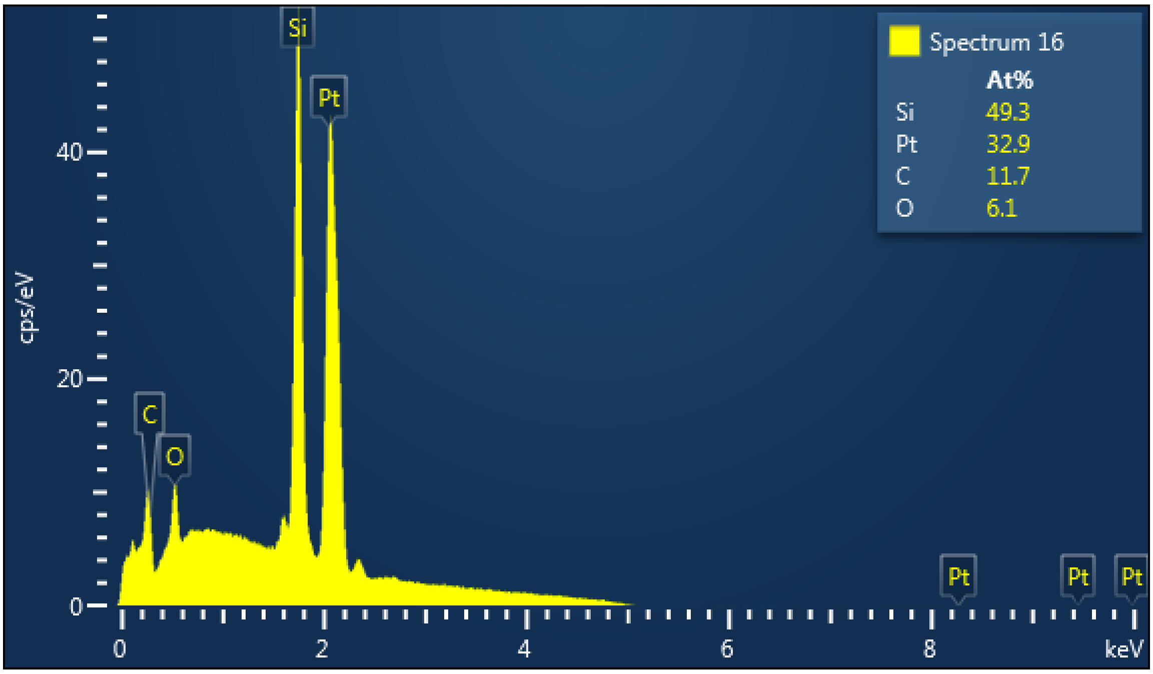
Spectrum 16
At%
Si 49.3
Pt 32.9
C 11.7
O 6.1
Si
Pt
C
O
Pt
Pt
Pt
cps/eV
0
20
40
0
2
4
6
8
keV

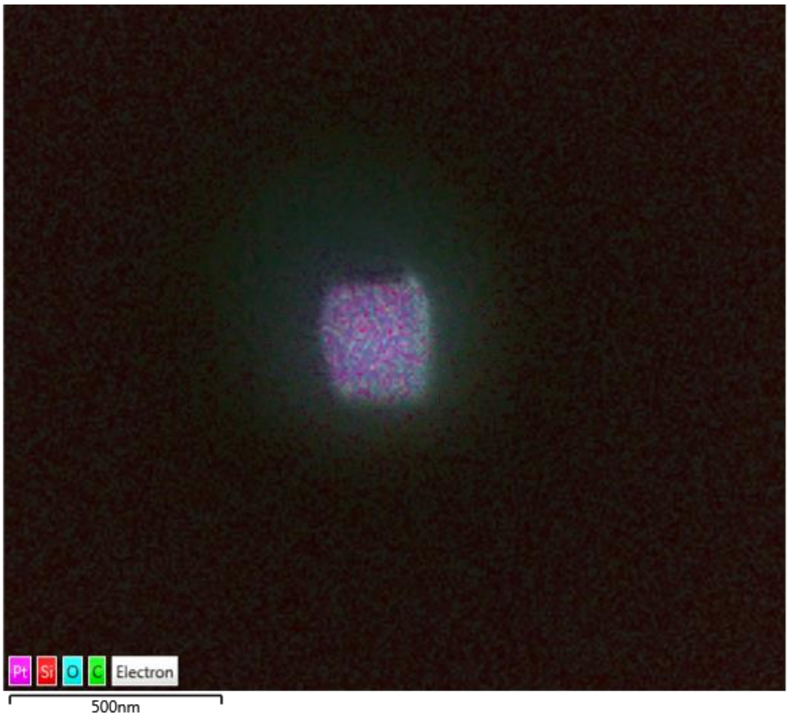
Pt
Si
O
C
Electron
500nm

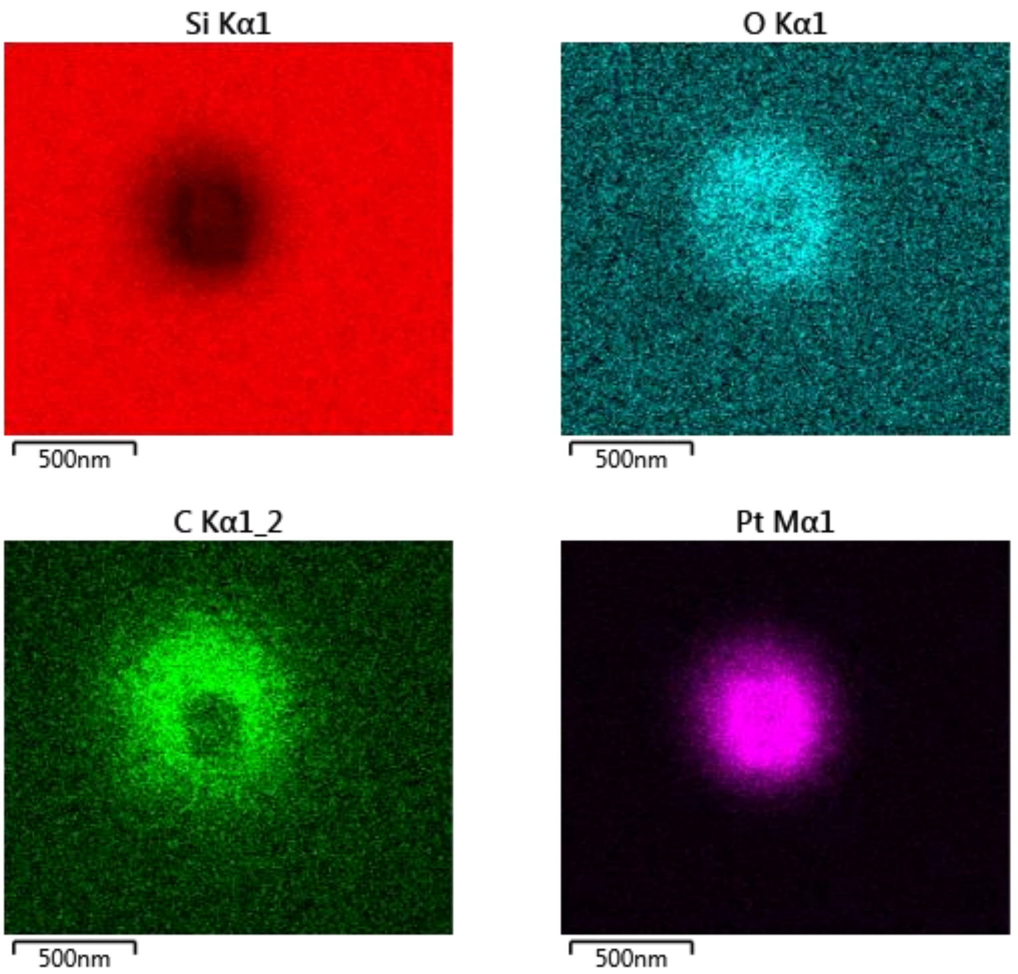

Figure S18: Top: EDX spectrum of Pt deposit **1d**, middle: EDX layered image (SEM image + EDX maps), bottom: separate EDX maps for the detected elements.

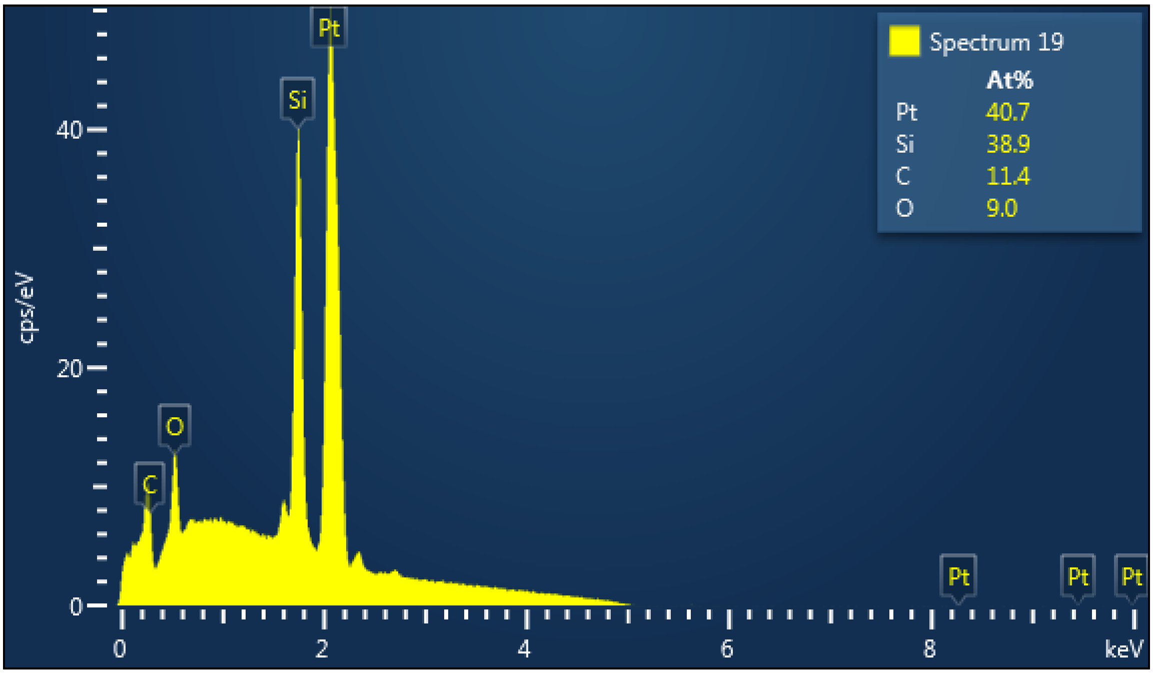
Pt
Si
O
C
Pt
Pt
Pt
Spectrum 19
At%
Pt 40.7
Si 38.9
C 11.4
O 9.0
cps/eV
40
20
0
0
2
4
6
8
keV

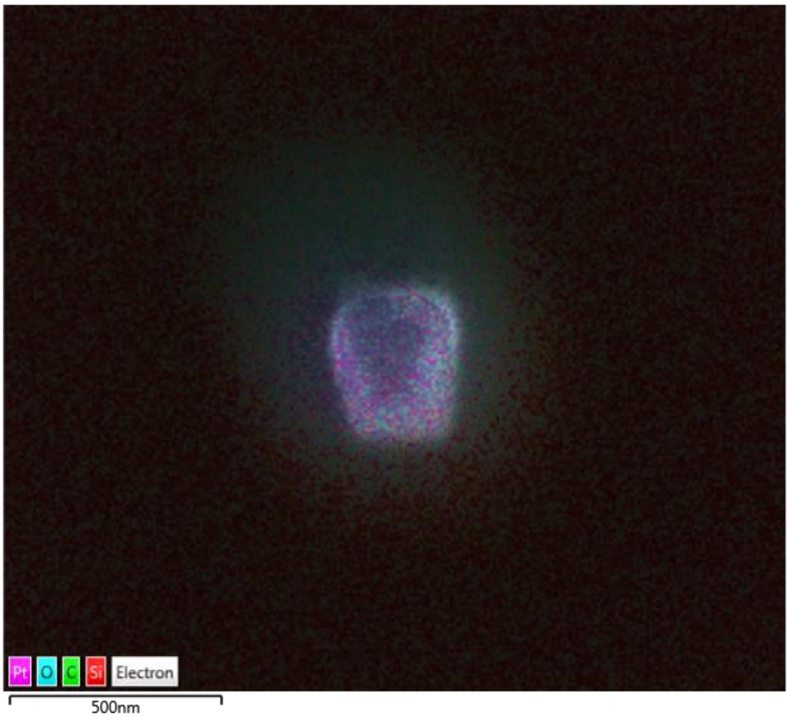
Pt
O
C
Si
Electron
500nm

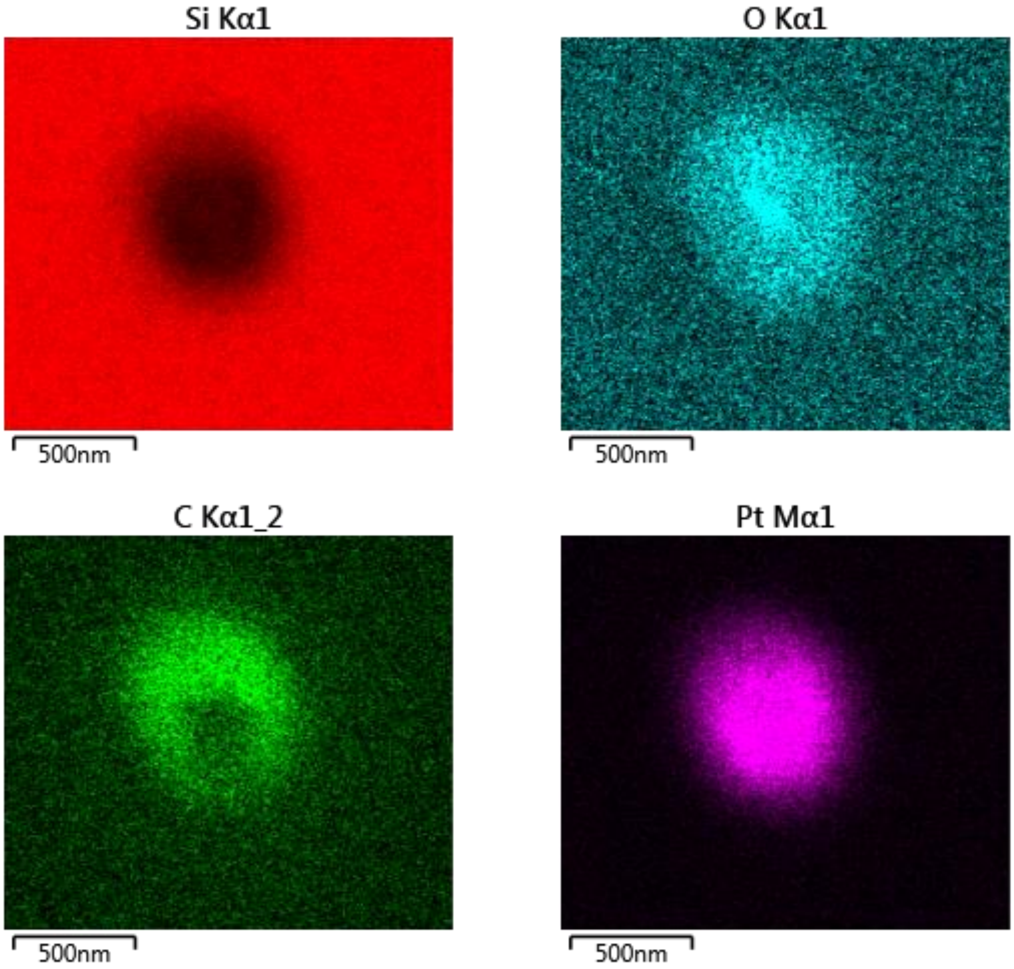

Figure S19: Top: EDX spectrum of Pt deposit **1e**, middle: EDX layered image (SEM image + EDX maps), bottom: separate EDX maps for the detected elements.

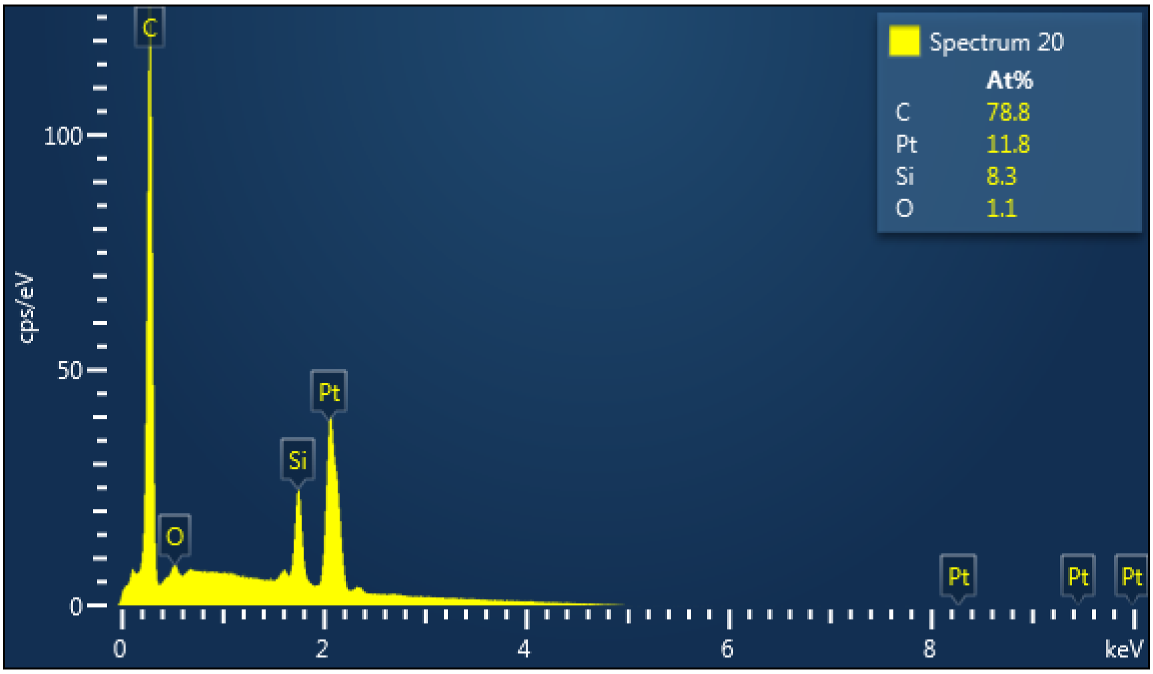
Spectrum 20
At%
C 78.8
Pt 11.8
Si 8.3
O 1.1
cps/eV
100
50
0
C
O
Si
Pt
Pt
Pt
Pt
0
2
4
6
8
keV

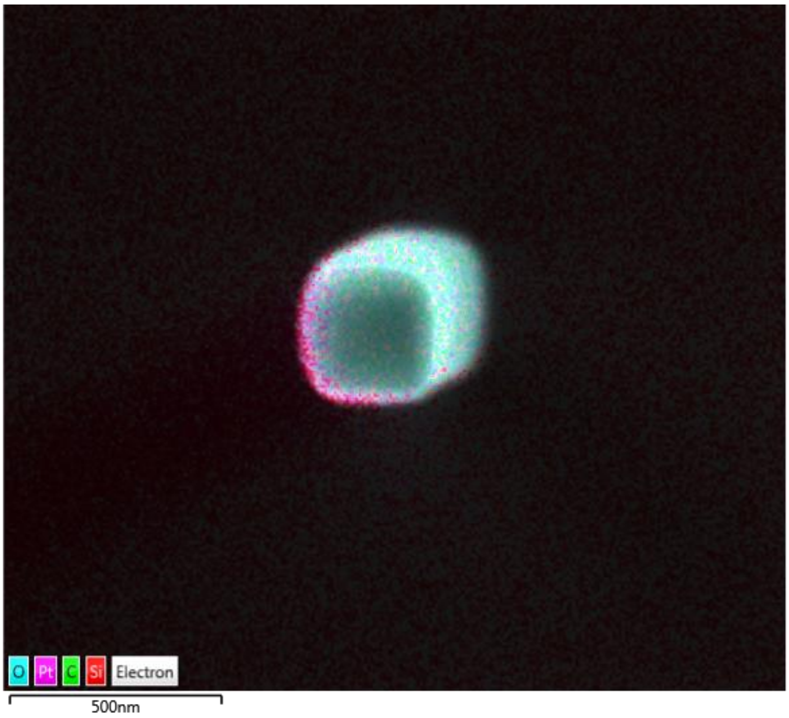
O
Pt
C
Si
Electron
500nm

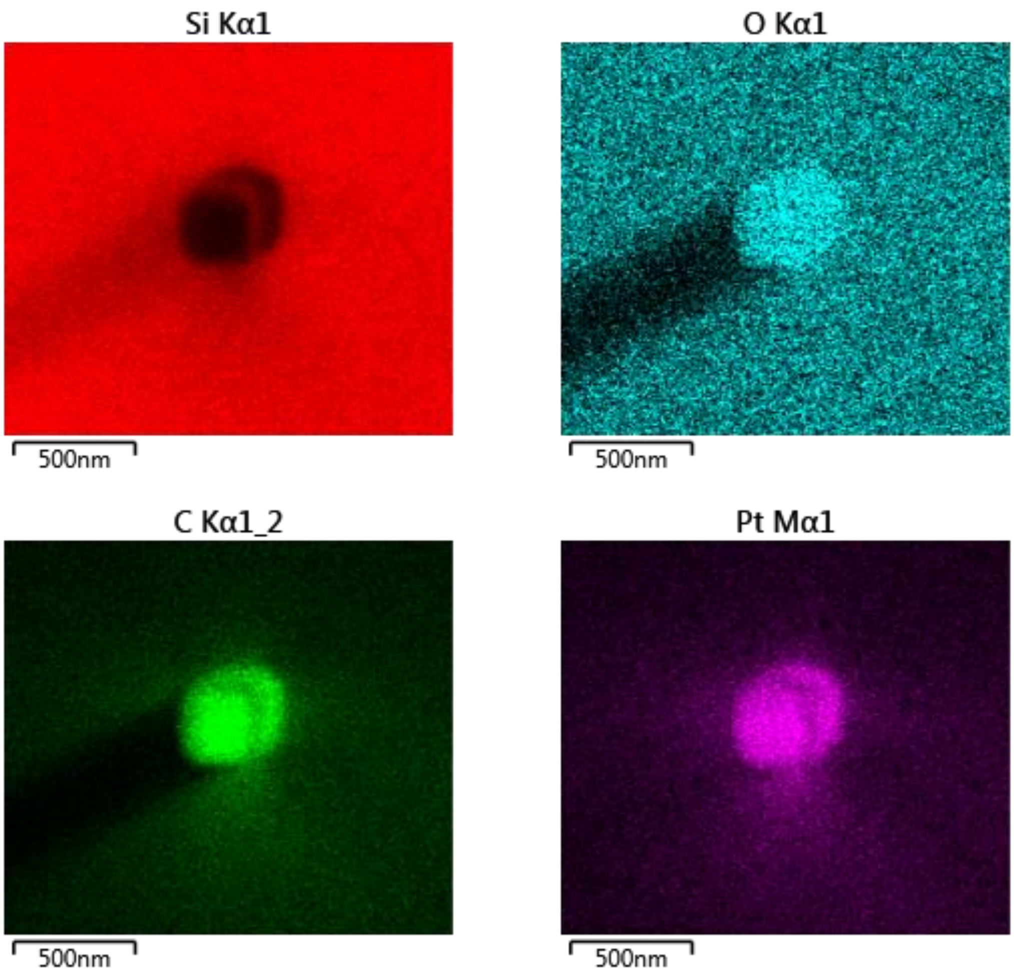

Figure S20: Top: EDX spectrum of Pt deposit **1f**, middle: EDX layered image (SEM image + EDX maps), bottom: separate EDX maps for the detected elements.

SEM-EDX and TEM-EDX comparison of a purified platinum deposit

In this section, the deposit analysed by SEM-EDX and TEM-EDX presented in the main manuscript as **1g** is described more extensively. To compare the SEM-EDX and TEM-EDX, line-scan EDX was used as the main method of comparison. If we define the plane of the Si substrate as the *xy* plane, the growth of the deposit happens in the *z* direction. During SEM EDX, the sample can be scanned only in the *xy* plane. The SEM-EDX line scan of **1g** and its composition plot along the *x* axis direction is presented here as Figure S21. A lamella of the sample was produced for TEM-EDX analysis. The lamella was cut roughly at the same position as the line scan shown in Figure S21. The TEM-EDX line scan was performed in the *z* direction of the sample, as shown by the green arrow in Figure S22. The subsequent composition plot represents the atom % obtained from that line scan. The EDX map of **1g** is presented combined in a single map in the main manuscript, while here it is presented as separate maps for each selected element in Figure S23. 1g: 250 × 250 nm^2, 1 µs dwell time, 18 keV, 1.7 nA, 4 nm pitch, 150000 passes, and pressure $5.69 \cdot 10^{-5}$ mbar.

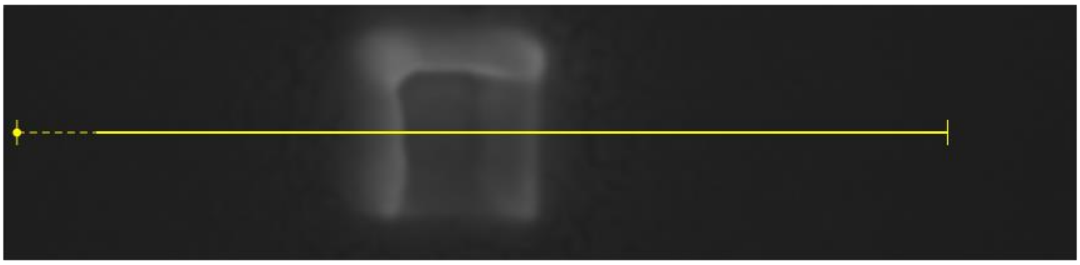

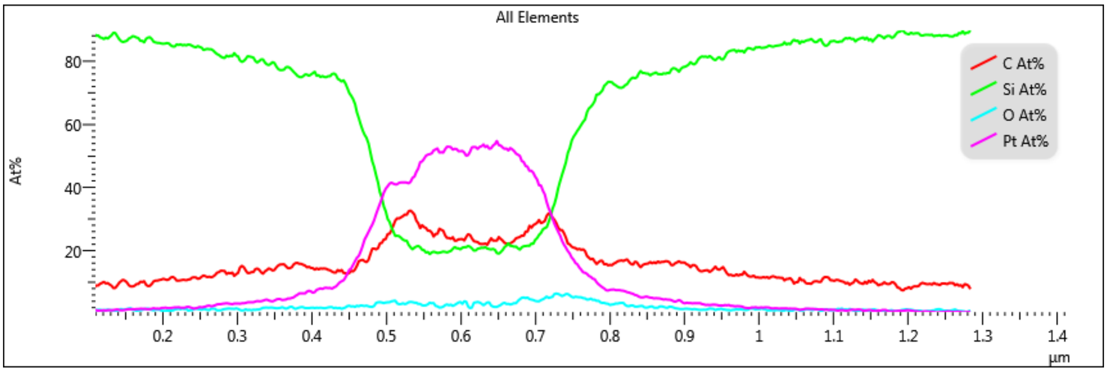

Figure S21: SEM image and composition values of the line scan EDX spectrum of **1g**. The yellow line defines the line-scan range.

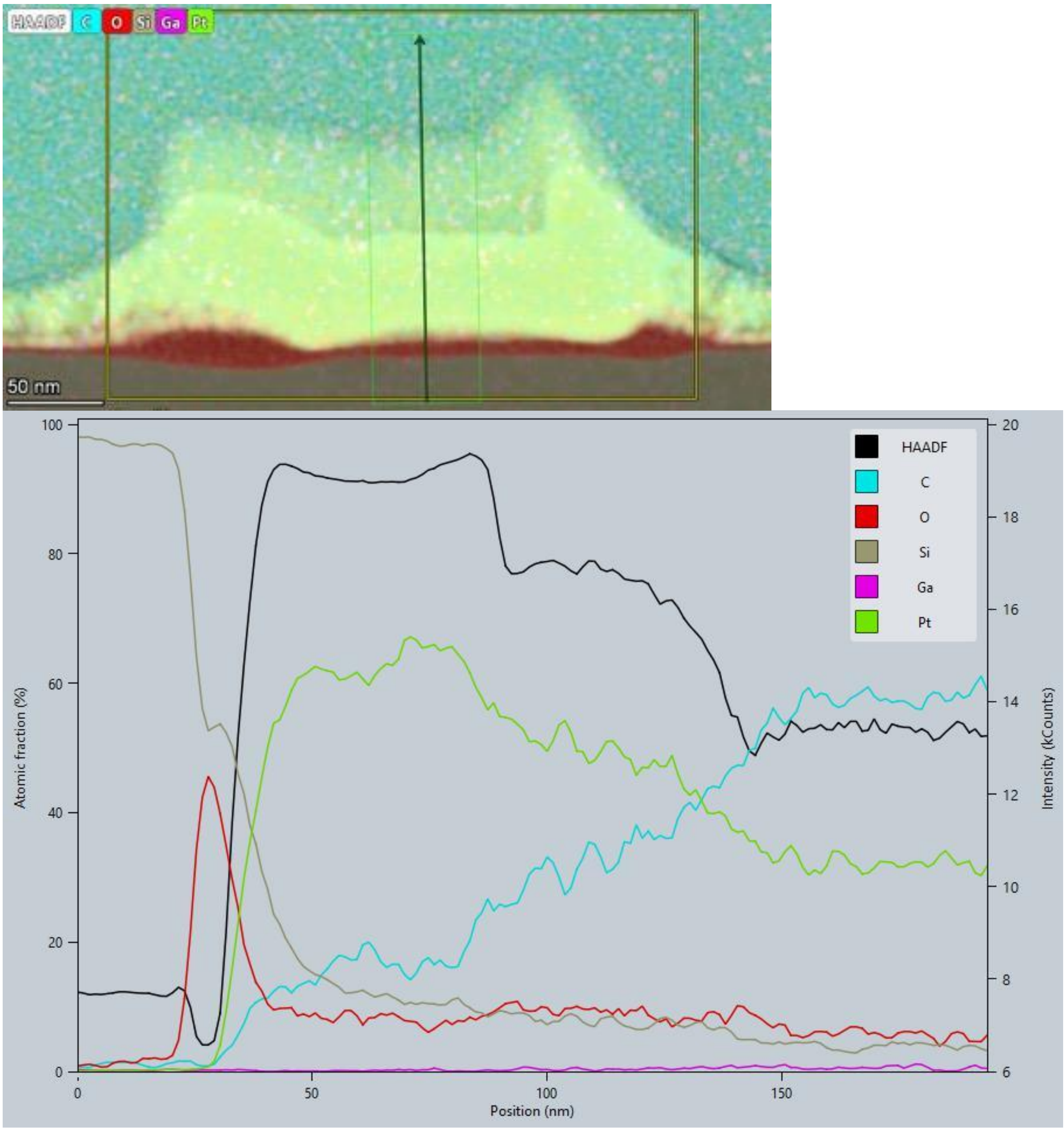

Figure S22: TEM-EDX map spectra of the lamella of deposit **1g**; the green arrow indicates the direction and range of the line-scan TEM-EDX. TEM-EDX composition values of the line EDX spectrum of the lamella of deposit **1g** are displayed below.

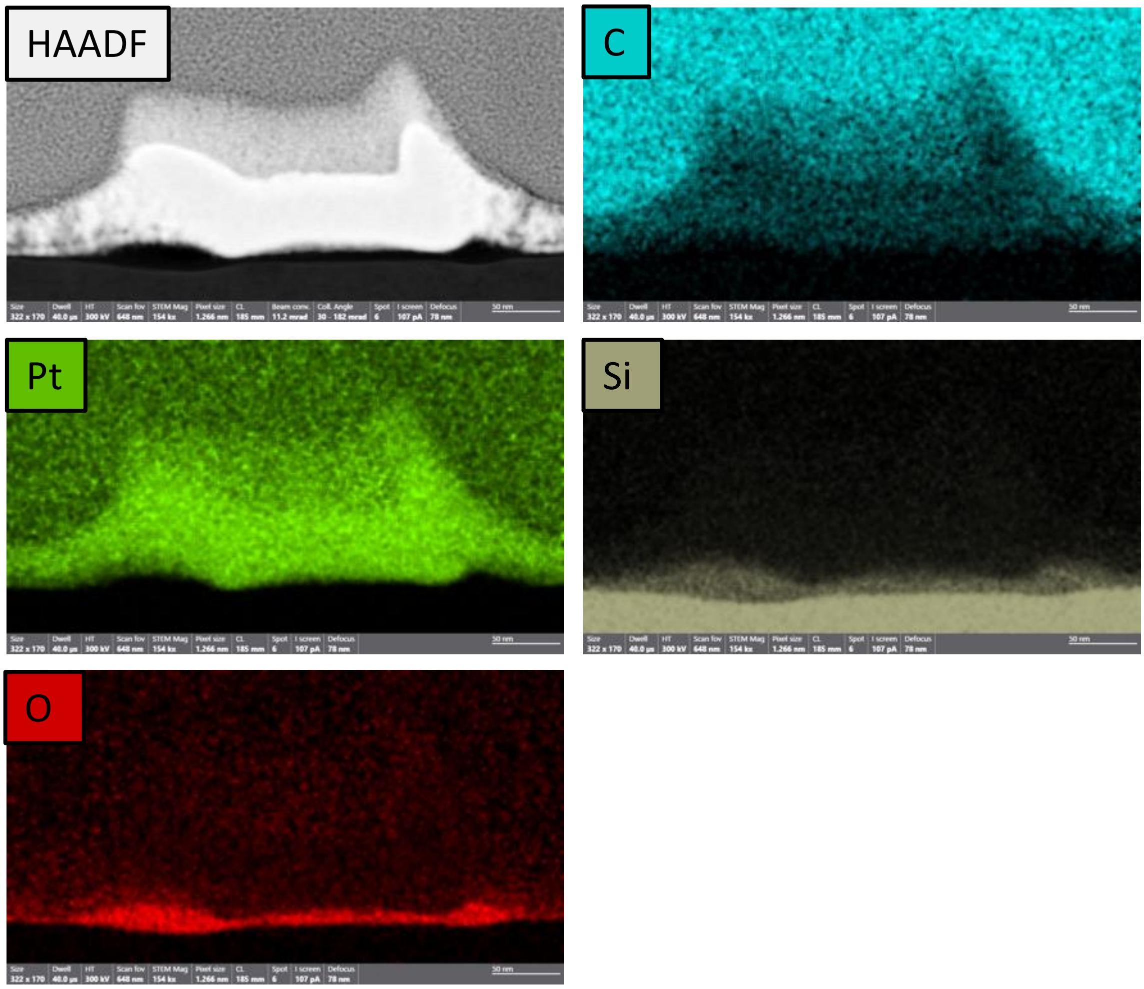

Figure S23: HAADF image (greyscale) and TEM-EDX maps (C, Pt, Si, and O) of deposit **1g**.

Platinum deposit purification: process investigation

The patterning conditions were: **2a–2f:** 150 × 150 nm^2, 10 µs dwell time, 5 keV, 4 nm pitch, 1, 5, 10, 50, 100, 500, 1000, 5000, and 10000 passes, 2.3 nA (**2a–2c**), 0.54 nA (**2d–2f**). **2a**, **2b**, **2d**, and **2e** are performed at a pressure of circa $5 \cdot 10^{-5}$ mbar, **2c** and **2f** at circa $1 \cdot 10^{-6}$ mbar. **2g**: 1 µm line, 5 keV, 0.54 nA, 1 µs dwell time, 4 nm pitch, 100000 passes, pressure circa $5 \cdot 10^{-5}$ mbar. BSE imaging can be used to obtain compositional information on the sample. Heavier elements are brighter in BSE imaging, making the identification of platinum in deposits **2a, 2c, 2d** and **2f** (Figure S24) straightforward.

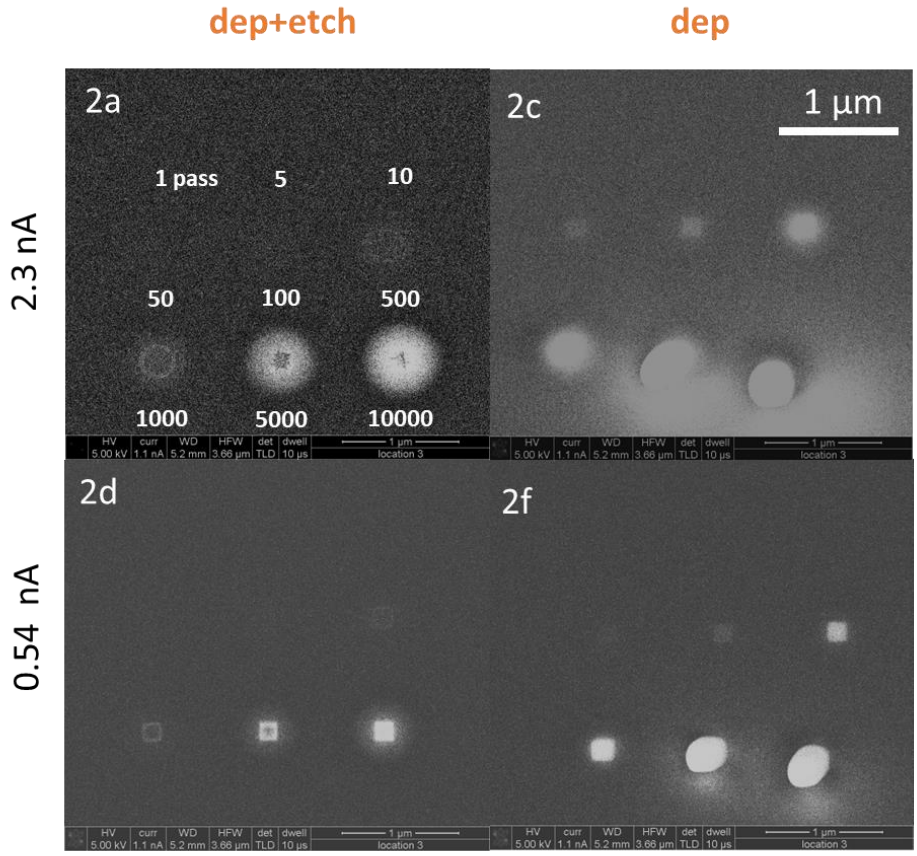

Figure S24: SEM BSE images of **2a**, **2c**, **2d**, and **2f**.

References

1. Mehendale, S.; Mulders, J. J. L.; Trompenaars, P. H. F. *Nanotechnology* **2013,** *24,* 145303. doi:10.1088/0957-4484/24/14/145303
2. Geier, B.; Gspan, C.; Winkler, R.; Schmied, R.; Fowlkes, J. D.; Fitzek, H.; Rauch, S.; Rattenberger, J.; Rack, P. D.; Plank, H. *J. Phys. Chem. C* **2014,** *118,* 14009–14016. doi:10.1021/jp503442b
